# Testing antimicrobial peptides inhibiting protein synthesis in living *E. coli* and *K. pneumoniae* using bio-orthogonal non-canonical amino-acid tagging

**DOI:** 10.3389/fmicb.2025.1713216

**Published:** 2025-12-16

**Authors:** Luigi de Pascale, Agnese D’Amore, Adriana Di Stasi, Martino Morici, Sabrina Pacor, Alessandro Tossi, Thuy Duong Pham, Attilio Fabbretti, Daniel N. Wilson, Mario Mardirossian, Marco Scocchi

**Affiliations:** 1 Department of Life Sciences, University of Trieste, Trieste, Italy; 2 Institute for Biochemistry and Molecular Biology, University of Hamburg, Hamburg, Germany; 3 School of Biosciences and Veterinary Medicine, University of Camerino, Camerino, Italy

**Keywords:** antimicrobial peptide, protein synthesis, click-chemistry, drug discovery, proline-rich, amino acid analogue

## Abstract

**Introduction:**

Antimicrobial peptides (AMPs) inhibit bacteria through diverse mechanisms of action. While many AMPs exert their effects by interacting with and damaging bacterial membranes, a growing subset has been shown to target intracellular processes, such as protein synthesis. In this study, we assessed the suitability of the bio-orthogonal non-canonical amino acid tagging (BONCAT) technique to investigate the mechanism of action on protein synthesis of proline-rich antimicrobial peptides (PrAMPs) in living Gram-negative bacteria.

**Methods:**

By combining BONCAT with flow cytometry to evaluate membrane integrity using propidium iodide assay we first validated the effective inhibitory activity of PrAMPs on protein synthesis leaving intact the bacterial inner membrane in both *Escherichia coli* and *Klebsiella pneumoniae*, thus extending beyond *E. coli* as the sole model organism.

**Results:**

We demonstrated that this approach can discriminate between AMP affecting protein synthesis from those with membranolytic activity. We showed that different PrAMPs, significantly reduced the protein synthesis in 10 min, suggesting a very rapid inhibition kinetics. Furthermore, unlike chloramphenicol, PrAMPs demonstrated a prolonged inhibitory effect on protein synthesis even after the peptides were removed from the medium, suggesting a long-lasting post-antibiotic effect.

**Discussion:**

We therefore demonstrated the validity of BONCAT as a tool for studying the molecular mechanisms of PrAMPs and we suggest that, in principle, this method may be extended also to other types of antimicrobial compounds to provide new insights into their mode of action on living bacteria.

## Graphical abstract

**Figure:**
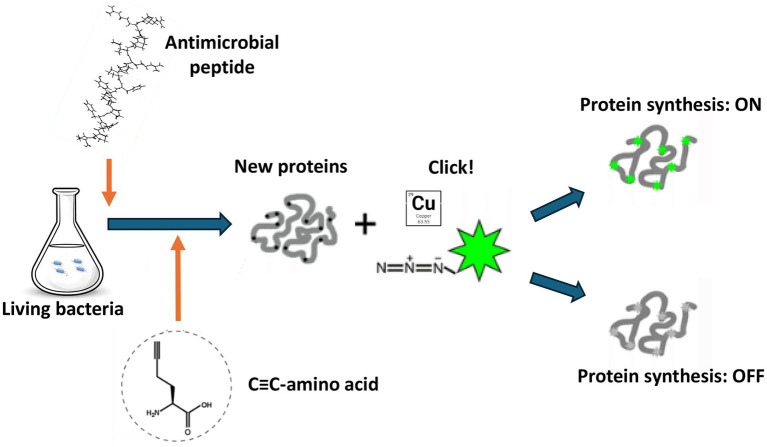

## Introduction

Antimicrobial peptides (AMPs), also referred to as host defence peptides, have gained significant attention as potential therapeutic agents for combating infectious diseases (Gera et al., 2022; Magana et al., 2020; Yang et al., 2025) and as innovative immunomodulatory treatments (Mahlapuu et al., 2020). They exhibit strong antibacterial, antiviral, and antifungal activities (Wang, 2014) and are prevalent across multicellular eukaryotes. In fact, most plants and animals express a wide array of AMP genes, particularly in epithelial tissues, and in response to infections (Lazzaro et al., 2020).

While it was once customarily held that AMPs primarily exerted their effects through membrane disruption and permeabilization (Benfield and Henriques, 2020; Travkova et al., 2017), subsequent research has revealed multiple additional mechanisms of action other than disruption of the bacterial envelope (Le et al., 2017; Scocchi et al., 2016; Zhang et al., 2021). Proline-rich AMPs (PrAMPs) are a notable subclass whose mode of action primarily relies on intracellular action with no morphological changes (Anderson et al., 2004; Shi et al., 1996). They can in fact cross cell membranes without damaging them (Podda et al., 2006) and inhibit protein synthesis, resulting in bactericidal activity (Graf and Wilson, 2019; Li et al., 2014; Welch et al., 2020). Other types of AMPs also act internally, for instance indolicidin, has been shown to significantly impact on bacterial DNA synthesis (Hsu et al., 2005), whereas microcin J25 (MccJ25) primarily inhibits RNA polymerase (Mukhopadhyay et al., 2004). Furthermore, other AMPs are suggested to drive the compaction of nucleic acids and modulate phase transitions (Sneideris et al., 2023).

PrAMPs are widespread, having independently evolved in many arthropods as well as in all families of the *Artiodactyla* order of mammals (Tossi et al., 2024), where they function as the active domains of cathelicidins (Scocchi et al., 2011; Welch et al., 2020). PrAMPs exhibit a narrower spectrum of activity compared to membranolytic AMPs, both *in vitro* and *in vivo*, being mainly directed at Gram-negative bacteria (Benincasa et al., 2010; Berthold et al., 2013; Runti et al., 2017) and without damaging host eukaryotic cells (Armas et al., 2021; Hansen et al., 2012; Li et al., 2014). Structural and biochemical studies on a representative number of insect and mammalian PrAMPs have shown that they enter the cytoplasm of *E. coli* by exploiting bacterial transporters (Krizsan et al., 2015; Mattiuzzo et al., 2007) without perturbation of cell membranes and then bind to the 50S subunit of bacterial ribosomes, thereby inhibiting protein synthesis (Graf et al., 2017). The binding site of all so far investigated PrAMPs is situated within the nascent polypeptide exit tunnel, although distinct inhibitory mechanisms may divide PrAMPs into two classes (I and II), according to the step of the protein synthesis that is impaired (Roy et al., 2015; Seefeldt et al., 2016, 2015).

Our understanding of the intracellular mechanism of action of AMPs, and specifically PrAMPs, has primarily been derived from functional studies (Krizsan et al., 2014; Mardirossian et al., 2020, 2014), structural studies performed using *in vitro* systems (Florin et al., 2017; Gagnon et al., 2016; Raulf et al., 2025; Seefeldt et al., 2016, 2015), and studies using radioactive tracers to treat bacteria (Mardirossian et al., 2018, 2014; Subbalakshmi and Sitaram, 1998). However, all of these studies have been conducted on, and are limited to, *E. coli* (Florin et al., 2017; Mardirossian et al., 2018; Taniguchi et al., 2016), while evidence has emerged suggesting that the mechanism of action of AMPs, including PrAMPs, can vary significantly across different bacterial species (Di Stasi et al., 2025; Oliveira Júnior et al., 2025). The complexity of these techniques and their inherent limitations hinder the assessment of protein synthesis inhibition in the development of peptide analogues with improved properties, discouraging evaluation studies on a large number of compounds and/or testing on a broad range of bacterial species.

Amino acid analogues were successfully employed about a decade ago to detect newly synthesized proteins within bacteria through a click chemistry technique known as Bioorthogonal Non-canonical Amino Acid Tagging (BONCAT) (Hatzenpichler et al., 2014). In this approach, bacterial cells are incubated with methionine analogues, such as L-homopropargylglycine (HPG) or L-azidohomoalanine (AHA), which carry azide or alkyne groups. Once these analogues are incorporated into newly synthesized proteins, they can be fluorescently labelled via a copper(I)-catalysed azide-alkyne cycloaddition (click chemistry) reaction with corresponding alkyne- or azide-labelled fluorophores. This allows for the visualization of cells actively engaged in protein synthesis through fluorescence (Lindivat et al., 2020) and, by extension, also highlights cells where protein synthesis is inhibited. However, despite BONCAT’s broad suitability for studying protein synthesis inhibitors, its application to Proline-Rich PrAMPs remained unexplored.

In this study, we evaluated BONCAT as a versatile tool for studying the inhibitory effects of PrAMPs on translation in Gram-negative bacteria. Using BONCAT coupled with flow cytometry, we monitored the reduced incorporation of fluorophore-labelled L-homopropargylglycine into newly synthesized proteins in *E. coli* and *K. pneumoniae* cells with non-compromised membranes. We analyzed three different PrAMPs in parallel with reference antibiotics possessing well-established molecular targets to validate the application utility in detecting protein synthesis inhibition by PrAMPs. Furthermore, we assessed the timing of inhibition and the post-antibiotic effects of the PrAMPs in *E. coli.* To demonstrate the method’s broad utility in mechanistic screening, we combined BONCAT with the PI-assay (Propidium Iodide assay), including colistin and a lytic AMP as controls, thereby establishing a tool for discriminating protein synthesis inhibition from membranolytic activity. Our results indicate that BONCAT is highly effective in investigating the mode of action of PrAMPs and could, in principle, be applied to other types of AMPs or antimicrobial compounds to assess their impact on protein synthesis in living bacteria.

## Materials and methods

### Peptides and antibiotics

All solid-phase synthesised peptides were purchased from NovoPro Bioscience (Shanghai, China) with a purity of > 95%. PMAP (12–31) was a generous gift from University of Padoa (Biondi et al., 2023). All the peptides were resuspended in 500 μL of 10 mM HCl and lyophilised three times to remove trifluoroacetic acid (TFA) as a counterion. They were then resuspended in sterile Milli-Q water and quantified spectrophotometrically at 214 nm and 280 nm (Ultraspec 2,100 pro, Amersham Bioscience). The molar extinction coefficients were calculated in accordance with Kuipers and Gruppen (2007). Colistin, chloramphenicol, rifampin and nalidixic acid were purchased from Sigma-Aldrich (USA). Chloramphenicol (CHL) and tetracycline (TET) were dissolved in ethanol (95% v/v) at concentrations of 3.5 mg/mL and 7.5 mg/mL, respectively. Rifampicin (RIF) was dissolved in dimethyl sulfoxide (DMSO, Sigma-Aldrich) at the concentration of 10 mg/mL. Nalidixic Acid (NDX) was dissolved in DMSO at a concentration of 2.5 mg/mL. Colistin (COL) was dissolved in sterile Milli-Q water at 2.5 mg/mL.

### Chemicals and solutions

The amino acid analogue homopropargylglycine (HPG) (Kiick et al., 2002) was purchased from Thermofisher Scientific: concentrated solution of 500 μM was prepared in Milli-Q water from a stock solution at 100 mM in DMSO. CuSO_4_ was purchased from Sigma-Aldrich (USA) and solved in Milli-Q water at 20 mM before being filtered (0.2 μm filter). Tris(3-hydroxypropyltriazolylmethyl) amine (THPTA) was purchased from Sigma-Aldrich (USA)and dissolved in 0.2 μm filtered Milli-Q water at 50 mM. Alexa Fluor azide 488 was purchased from Thermofisher Scientific and solved in DMSO at 10 mM. Sodium ascorbate and Aminoguanidine were both purchased from Sigma-Aldrich (USA) and dissolved in Milli-Q water at 100 mM before being filtered (0.2 μm filter). All the mentioned reagents were stored at −20 °C, except for HPG and copper sulfate, both of which were stored at 4 °C (the former screened from light exposure).

A premix solution was prepared by mixing 20 mM copper sulfate, 50 mM THPTA and 10 mM Alexa Fluor azide 488 in the ratio 20:40:1. The premix solution was always freshly prepared and incubated for 3 min in the dark at 25 °C before use. Propidium iodide, purchased from MERCK LIFE SCIENCE S.R.L, was dissolved in PBS at a concentration of 0.1 mg/mL, and stored in the dark at 4 °C.

### Bacteria strains and growth conditions

*E. coli* ATCC 25922, *K. pneumoniae* ATCC 700603 and ATCC 13883, *A. baumannii* ATCC 19606, *S. aureus* ATCC 25923, *P. aeruginosa* ATCC 27853, *E. cloacae* ATCC 13047 and *E. faecium* ATCC 19434 were purchased from American Type Culture Collection (ATCC) (Manassas, Virginia, USA) and from the Leibniz institute DSMZ (Braunschweig, Germany) and were stored at −80 °C as glycerol stock until use. M9 Minimal Salts Base 1x (M9 medium base) was prepared diluting sterilized BD Difco™ (New Jersey, U.S.) Dehydrated Culture Media M9 medium base 5x were prepared and stored at 4 °C for up to a month. All assays with bacteria were performed in 0.2 μm filtered M9 minimal salts supplemented with 0.2 μm filtered 2 mM MgSO_4_ and 0.2 μm filtered 5 mM glucose (supplemented M9 medium).

All bacterial cultures were grown aerobically overnight in a shaker at 130 rpm and 37 °C in supplemented M9 minimal salt medium with the addition of 1% (v/v) Mueller Hinton broth (MHB, Difco). The next day, the cultures were washed and resuspended in fresh supplemented M9 medium.

### Minimum inhibitory concentration (MIC) assay

Minimum inhibitory concentration (MIC) assay was performed as reported previously (Sola et al., 2020) An overnight culture of *E. coli* ATCC 25922 or *K. pneumoniae* ATCC 700603, prepared as described above was incubated at 37 °C until reaching an optical density of approximately 0.5–0.6 at 600 nm. The culture was then diluted in supplemented M9 medium at the concentration of 1 × 10^7^ CFU/mL. Each peptide, diluted at a starting concentration of 128 μM in supplemented M9 medium was added to the first column of wells of a 96-well round-bottom microplate (Sarstedt, Milan, Italy). Serially two-fold dilution (1:1) were performed across subsequent wells to a final volume of 50 μL. 50 μL of bacterial suspension (1 × 10^7^ CFU/mL) were then added to each well, to a final bacterial concentration in each well to 5 × 10^6^ CFU/mL per well. Untreated bacterial culture was used as growth control, while 100 μL of uninoculated supplemented M9 medium was used to verify the sterility of the medium. The plates were sealed with parafilm to minimize evaporation and incubated at 37 °C for 18 h. The minimum inhibitory concentration (MIC) was visually determined as the lowest peptide concentration in a well with no visible bacterial growth. Data are reported as the mode of at least three independent experiments.

### Bacteria grow rate assays

An overnight culture of *E. coli* ATCC 25922, prepared as described above, was diluted to a final concentration of 5 × 10^6^ CFU/mL and then incubated at 37 °C for 30 min in the presence or absence of each peptide or antibiotic at various concentrations, with a final volume of 1 mL. To measure the post-antibiotic effect (PAE) of antimicrobials, the compounds were removed from the supernatant by centrifugation (16,000 × g for 5 min) and bacteria were washed and resuspended in 1 mL of fresh supplemented MHB medium, to promote faster growth visible as increased absorbance. 200 μL of each sample was then transferred to a 96-well round-bottom microplate (Sarstedt, Milan, Italy), which was then placed in a Nanoquant Infinite-M200Pro plate reader with stable temperature at 37 °C. Each sample was surrounded by water-filled wells to reduce its own evaporation. Optical density at 600 nm was then recorded for each sample every hour for up to 18 h. PAE was calculated according to Holfeld et al. (2018) as the difference between the time needed for a cell culture incubated with an antibiotic substance to reach half-maximum absorbance of the control culture and the time that the control culture needed to reach half-maximum absorbance of the stationary phase. According to this definition, the PAE of each compound (expressed as time) was calculated based on the OD₆₀₀ value of its respective replicate, which corresponded to half of the maximum absorbance reached by the control culture in the stationary phase (at 18 h). The mean PAE values (PAE in hours) of each compound, were obtained by averaging the times from the three independent experiments.

### Homopropargylglycine incorporation into bacteria

We used a modified protocol from Lindivat et al. (2020). Briefly, overnight cultures of *E. coli* ATCC 25922 or *K. pneumoniae* ATCC 700603, prepared as described above, were diluted to a final concentration of 5×10^6^ CFU/mL and allowed to adjust for 30 min at 37 °C. The culture was then incubated at 37 °C in the dark for 10 or 30 min in the presence or absence of each peptide or antibiotic at various concentrations, with a final volume of 1 mL. At this point, only in certain specified experimental assays, the compounds were removed from the supernatant by centrifugation (16,000 × g for 5 min) and bacteria were washed and resuspended in fresh supplemented M9 medium. Then, 32 μL of a 500 μM homopropargylglycine (HPG) solution were added to each sample, resulting in a final concentration of 16 μM in a finale volume of 1 mL. Also included in each assay were two untreated controls containing only bacteria and bacteria treated with 16 μM HPG alone, respectively. All samples were then incubated at 37 °C in the dark for 30 min. After incubation, samples were washed once with 1X filtered Phosphate Buffered Saline (PBS) (centrifuged at 16,000 x g for 5 min) and resuspended in PBS. After washing, samples were fixed with formaldehyde (Sigma Aldrich, USA) to a final concentration of 3% in 1 mL for 1 h at 25 °C. Subsequently, the samples were centrifuged at 16,000 x g for 5 min and resuspended in 1 mL of PBS. If not analysed immediately, the fixed samples were stored at 4 °C prior to performing the click chemistry reaction. Samples were centrifugated at 16,000 x g for 5 min and bacteria cells were dehydrated by ethanol that was added sequentially to achieve a final concentration of 50% (v/v) (3 min of incubation), 80% (v/v) (3 min of incubation), and 95% (v/v) (3 min of incubation). After dehydration, the samples were centrifugated (16,000 × g, 5 min) and resuspended in PBS. For cell analysis by SDS-PAGE, the following variations were implemented: bacterial cells were diluted in supplemented M9 medium to a final density of ~ 1 × 10^9^ CFU/mL, and HPG was added to the samples at a final concentration of 160 μM. Bacteria were incubated with HPG for 0, 15, 30, 60, or 120 min at 37 °C in the dark.

### Cu(I)-catalysed alkyne- azide click chemistry reaction

The click reaction was carried out according to Rostovtsev et al. (2002). 15 μL of a pre-mix solution (prepared as described above) were added to each sample containing fixed bacteria that had incorporated HPG (30 μL of pre-mix solution in case of samples prepared for SDS-PAGE analysis), resulting in the following final concentrations in each sample: 0.1 mM copper sulfate, 0.5 mM Tris (3-hydroxypropyltriazolylmethyl)amine (THPTA), and 2.5 μM Alexa Fluor azide 488. Additionally, 50 μL of freshly prepared 100 mM sodium ascorbate and 50 μL of 100 mM aminoguanidine were added (100 μL in case of samples prepared for SDS-PAGE analysis), bringing the final volume to 1 mL. The samples were gently inverted once to mix and incubated for 30 min in the dark at 25 °C. Finally, each sample was washed with PBS once (centrifuged at 16,000 × g for 5 min) and resuspended in 1 mL PBS.

### Bacteria analyses by flow cytometry

An overnight culture of *E. coli* ATCC 25922, or of *K. pneumoniae* ATCC 700603, prepared as described above, was diluted to a final concentration of 5 × 10^6^ CFU/mL and allowed to adjust for 30 min at 37 °C under shaking. Membrane permeabilization of bacteria was then assessed by incubating bacteria at 37 °C, in a final volume of 1 mL, with varying concentrations of each peptide or antibiotic used for the BONCAT assay. 5 min before the ending of the selected incubation time (40 min), propidium iodide probe was added to the samples to a final concentration of 10 μg/mL before analysis using a flow cytometer. A positive control consisted of *E. coli* cells dehydrated with ethanol (as described above), while untreated cells were used as negative control. The fluorescence of untreated cells was used to establish the gating for positive PI-stained cells. Fixed bacterial cells, subjected to the click chemistry reaction to link the Alexa Fluor azide 488 probe, were analysed by flow cytometry and, among the gates-selected ones, only those that tested positive for Propidium Iodide, and thus permeabilized, were considered for the calculation of the mean fluorescence intensity (MFI) of the Alexa Fluor azide 488.

Samples were analyzed by an Attune NxT^®^ (ThermoFisher) equipped with a blue laser (488 nm) and photomultiplier tubes (PMTs) BL1 (525/50) and BL3 (695/40) to collect fluorescence. All parameters were set in logarithmic scale and the signals were recorded as Area. Forward scattering (FSC) was set to 300 mV, side scattering (SSC) to 350 mV and thresholds to 0.2 for FSC and 0.5 for SSC. The compensation matrix was performed according to the tool software. Hierarchical gates and acquisition flow rate (200 μL/min) were set to avoid coincident events. The files were saved as FCS 3.1 and analysed by FCS Express *De Novo* software v7. Flow cytometry data are presented as means ± SD from three independent experiments (*n* = 3).

### Analysis of HPG incorporation into bacteria by SDS PAGE

At the end of the click-chemistry incubation, each sample was washed with PBS once (centrifuged at 16,000 × g for 5 min) and resuspended in 50 μL volume, consisting of 37.5 μL of Milli-Q water and 12.5 μL of loading buffer 4 × (0.125 M Tris-Cl, 4% SDS, 20% v/v glycerol, 0.2 M DTT, 0.02% bromophenol blue, pH 6.8), and stored at −20 °C until use. Prior to analysis, samples were thawed, incubated on a thermoblock at 90 °C for 10 min and then centrifuged. 15 μL of the supernatant were loaded into the wells of a 4% polyacrylamide stacking gel. Protein samples were analysed by SDS-PAGE using a standard Laemmli Tris-glycine system. Electrophoresis was conducted at 200 V in Tris-glycine-SDS running buffer until the bromophenol blue band reached the gel bottom. After separation, the gel was scanned using the ChemiDoc system (BIO-RAD) selecting the appropriate channel for Alexa Fluor azide 488 to detect the fluorescence signal of the probe. Subsequently, the gel was stained overnight with Coomassie Brilliant Blue R-250 and destained using 10% (v/v) acetic acid in deionized water. The signal intensity of Coomassie Brilliant Blue R-250 was detected using ChemiDoc system at the appropriate channel for the dye. The ImageJ processing program was used to quantify and normalize the Alexa Fluor azide 488 fluorescence signals of each sample with the respective signal relative to Coomassie Brilliant Blue R-250. The results obtained were then reported in this paper in the form of a percentage compared with a control sample treated with HPG for the maximum time we tested (120 min), considered as 100%.

### ESKAPEs ribosome preparation

The ribosomes used in this study were purified as described previously (Di Stasi et al., 2025). Briefly, to purify the 70S ribosomes of ESKAPE pathogens, the bacterial strains *E. faecium* ATCC 19434 *S. aureus* ATCC 25923, *K. pneumoniae* ATCC 13883, *A. baumannii* ATCC 19606, *P. aeruginosa* ATCC 27853 and *E. cloacae* ATCC 13047 were grown in 2 L of Luria-Bertani (LB) medium, at 37 °C, until the mid-log phase was reached. After centrifugation at 7,000 rpm (12,000 × g) for 15 min at 20 °C (JLA rotor 8.1000), the bacterial pellet was resuspended in 50 mL of saline (0.9% w/v NaCl) and re-centrifuged at 15,000 rpm (27,000 × g) for 10 min at 20 °C (JA rotor 25.50). The bacterial pellet was resuspended in 10 mL of Nirenberg Buffer (NB) (10 mM Tris-HCI pH7.7, 60 mM NH4 Cl, 10 mM Mg acetate). The cells were then stored in liquid nitrogen at −80 °C and then thawed at the time of use. After thawing, the cells were subjected to sonication (Misonix 3,000) to induce lysis and dithiothreitol (DTT) was added at a final concentration of 1 mM with subsequent centrifugation at 15,000 rpm (18,000 × g) for 30 min at 4 °C to purify the cell extract. The supernatant was subsequently ultracentrifuged at 80,000 rpm (320,000 × g) for 2 h at 4 °C (S80-AT3 rotor) to resuspend the ribosomal pellet in NB with 1 mM DTT. Finally, the solution was cleared of the last remaining impurities by centrifugation at 15,000 rpm (18,000 × g) for 15 min at 4 °C, and the ribosomes subjected to quantification by Nanodrop (A_260_). All buffers mentioned in this paragraph were used after being filtered (MWCO = 0.2 μm).

### *In vitro* translation assay

The *in vitro* translation assay was performed by using the *Δ* ribosome from the *E. coli* PURExpress system New England Biolabs (NEB), as reported previously (Di Stasi et al., 2025; Raulf et al., 2025). Purified ESKAPE ribosomes, obtained as described in the previous paragraph, were used to replace *E. coli* ribosomes in the PURExpress kit by inserting 1 μL of their suspensions (about 8 pmol of 70S subunit) into 5 μL of PURExpress reaction mixture with the addition of 1 μL of antibiotics. Each sample contained 16 ng/μL of mRNA for firefly luciferase (Fluc) transcribed *in vitro* by T7 polymerase (Thermo Fisher Scientific) from a pIVEX-2.3MCS vector containing the Fluc gene. The reaction mixture was incubated 2 min at 32 °C (with stirring at 600 rpm) before mRNA addition, then incubated at 32 °C for 30 min (600 rpm). Each reaction was stopped by the addition of 5 μL of kanamycin (50 mg/mL) and transferred to the wells of a 96 microplate (Greiner Lumitrac, non-binding, white, chimney plates). Finally, 40 μL of luciferase assay substrate solution were added to the wells (Promega, E1501), and the luminescence measured using a plate reader (Tecan Infinite 200 PRO). The negative control of the test was nuclease-free water in place of the antibiotic, whose values were used to normalize the absolute luminescence values of the samples. This experimental assay was conducted in triplicate, using individually prepared reaction mixtures.

### Statistical analysis

The results of histograms presented in this work are shown as a percentage compared with the fluorescence of the untreated positive control (incubated with HPG and Alexa Fluor azide 488). Data are the mean ± SD of at least three experiments (*n* ≥ 3). *p < 0.05, **p < 0.01, ***p < 0.001 compared with the untreated sample (one-way ANOVA test).

## Results

**Figure 1:**
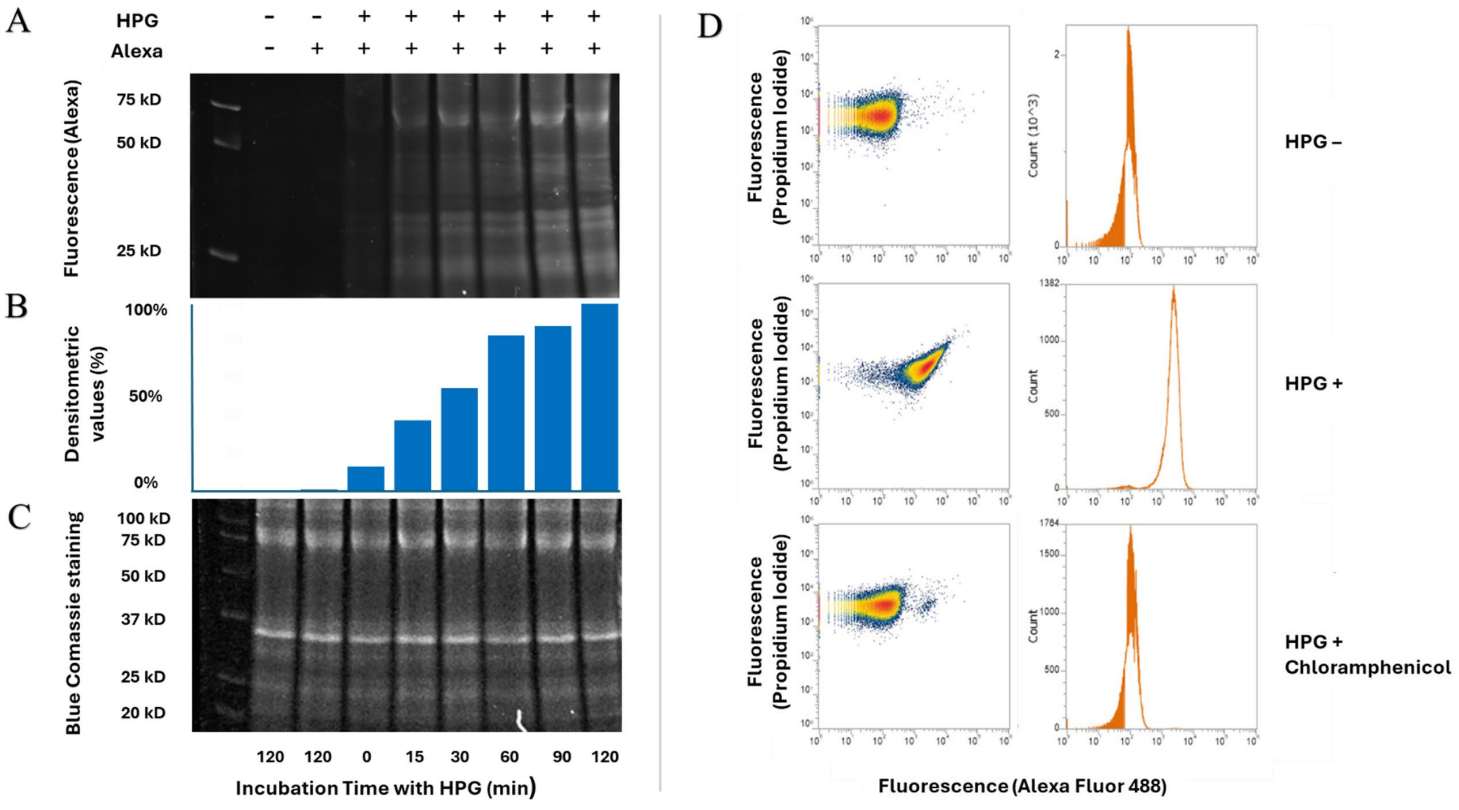
Incorporation of HPG in growing *E. coli* ATCC 25922. **(A–C)** SDS page of proteins extract from *E. coli* ATCC 25922 treated with homopropargylglycine (HPG) from 0 min up to 120 min. Proteins detected by fluorescence given by the linked Alexa 488 fluor probe **(A)**; densitometric analysis of fluorescent protein bands **(B)**; and proteins visualized by Coomassie blue staining **(C)**. **(D)** Flow cytometric analysis of *E. coli* cells treated with Alexa Fluor azide, HPG or HPG in the presence of chloramphenicol. Cells were pre-incubated for 10 min with chloramphenicol, followed by a 30-min incubation with the amino acid analogue HPG. After fixation, samples underwent a click chemistry reaction with the fluorescent probe Alexa 488 to label newly synthesized proteins. The data are representative of three replicate experiments with highly similar results.

To evaluate the potential inhibitory effects of antibacterial peptides on protein synthesis, we established a protocol based on the BONCAT method (Hatzenpichler et al., 2014; Lindivat et al., 2020). First, we validated the efficient incorporation of the methionine analogue, homopropargylglycine (HPG), into newly synthesized proteins under the selected bacterial growth conditions. *E. coli* ATCC 25922 cells were incubated for up to 120 min in M9 salts medium supplemented with 2 mM MgSO_4_ and 5 mM glucose, containing HPG but lacking any external amino acid source. Growth was monitored during this period (see Supplementary Figure S1). *E. coli* cells were then fixed and underwent a click chemistry reaction to specifically label HPG-containing proteins with an Alexa Fluor-azide probe. Subsequent SDS-PAGE analysis confirmed that bacterial proteins became fluorescent upon treatment, with a detectable signal appearing after only 15 min of incubation with HPG and increasing up to 120 min (Figures 1A–C). No fluorescence was observed in samples not treated with HPG (Figures 1A–C). We also analysed the HPG incorporation in proteins using flow cytometry (Figure 1D). To ensure optimal penetration of the fluorescent probe in bacteria, enabling therefore its binding to newly synthesised proteins, we confirmed that the cell fixation protocol enabled a high level of cell permeabilization to PI (Supplementary Figure S2). Additionally, we verified that the HPG concentration used did not affect bacterial growth (Supplementary Figure S3).

**Figure 2:**
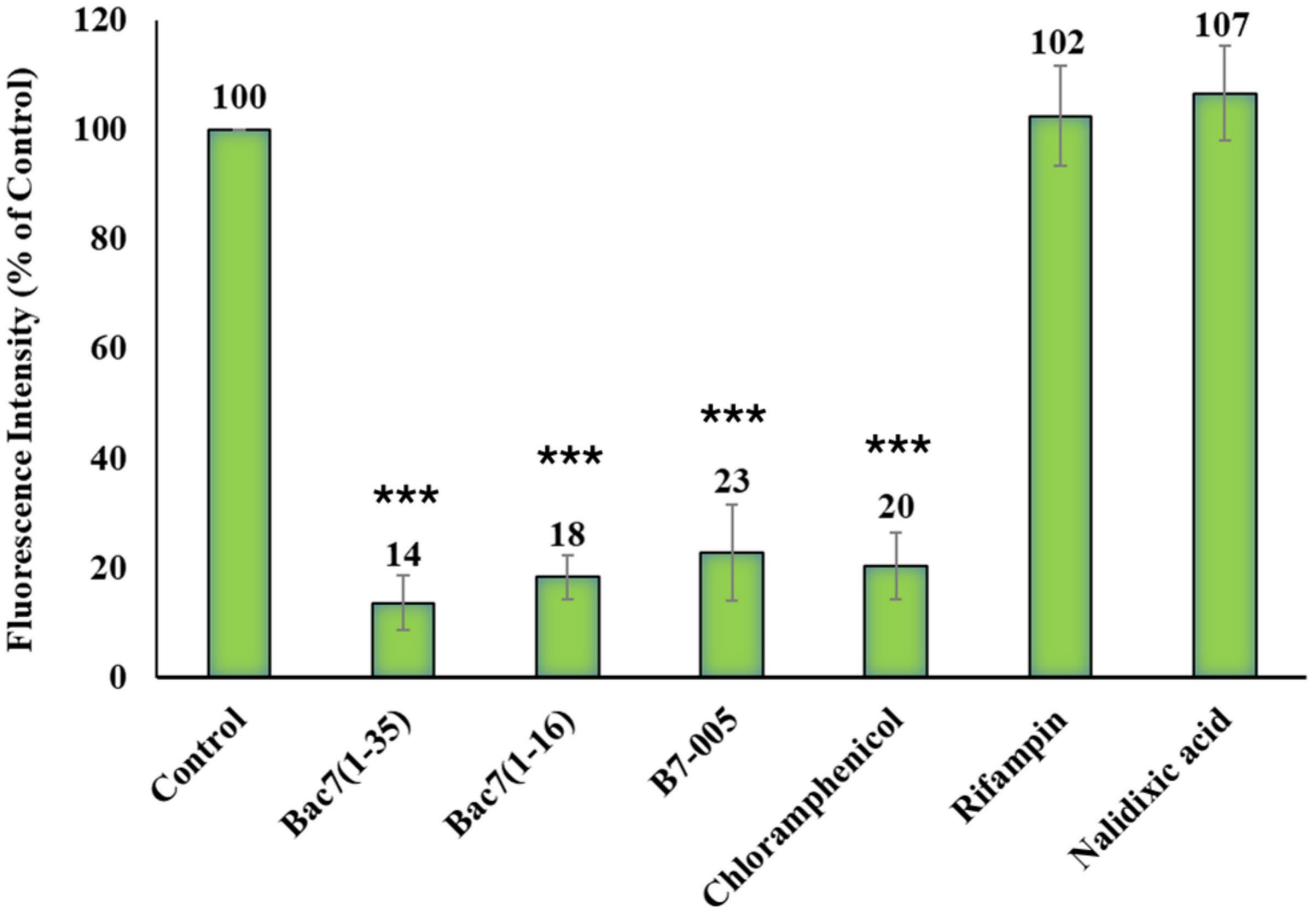
Flow cytometry analysis of HPG incorporation in *E. coli* cells treated with PrAMPs or antibiotics. Cells were pre-incubated for 10 min with the indicated compounds at their respective MICs reported in Table 1, followed by a 30-min incubation with the amino acid analogue HPG. After fixation, samples underwent a click chemistry reaction with the fluorescent probe Alexa 488 to label newly synthesized proteins. Fluorescence was measured and expressed as a percentage relative to untreated cells (Control) set as 100%. Data represent the mean and standard deviation of at least three independent experimental results (*n* ≥ 3). ***p < 0.001 compared with the untreated sample (one-way ANOVA test).

Next, we investigated the effects of PrAMPs on protein synthesis, monitoring HPG incorporation in cells treated with a panel of antimicrobials. This panel included three different proline-rich peptides (PrAMPs): Bac7(1–35), Bac7(1–16), and B7-005, all previously shown to inhibit *E. coli* protein synthesis (Mardirossian et al., 2020, 2014; Seefeldt et al., 2016). For comparison, we also tested an antibiotic known to inhibit protein synthesis (e.g., chloramphenicol, CHL), and another two with different cellular targets (e.g., rifampin, RIF, and nalidixic acid, NDX).

Before running these assays, we determined the MICs of all these compounds under the conditions to be used for BONCAT experiments, i.e., in the supplemented M9 salts medium and using 5 × 10^6^ CFU/mL *E. coli* cells (Table 1). Subsequently we confirmed the PrAMPs have not membrane-permeabilizing activity at their respective MICs, under the same specific experimental conditions, using flow cytometric analysis after incubating with PI (Table 1).

**Table 1:** Antimicrobial activity (MIC) and cell membrane permeabilization assay of different antimicrobial peptides and antibiotics against 5 × 10^6^ CFU/mL *E. coli* ATCC 25922 and *K. pneumoniae* ATCC 700603 in supplemented M9 medium.

Compound	*E. coli* ATCC 25922		*K. pneumoniae* ATCC 700603	
	MIC (μM) *	Membrane permeabilization (% PI +cells) **	MIC (μM)	Membrane permeabilization (% PI +cells)
Bac7(1–35)	1	<1%	4	<1%
Bac7(1–16)	1	<1%	4	<1%
B7-005	2–8	<1%	4	<1%
PMAP(12–31)	128	38%	n.d	n.d
COL	16	1%	16	n.d
RIF	2	<1%	16	<1%
NDX	16	<1%	n.d.	n.d.
CHL	32	<1%	n.d.	n.d.
TET	n.d.	n.d.	32	<1%
Ethanol	n.d.	78%	n.d.	96%

* Minimum Inhibitory Concentrations (MICs). MIC assays were performed using bacteria at an initial inoculum of 5×10^6^ CFU/mL. The MIC was defined as the lowest antimicrobial concentration showing no visible bacterial growth after 18 h of incubation at 37 °C. Values represent the mode MIC from at least three independent experiments (*n* ≥ 3). ** Cell permeabilization was assessed by incubating bacteria (5 × 10^6^ CFU/mL) with each compound at its respective MIC for 40 min. Results are expressed as the percentage of PI-positive cells relative to the total bacterial count. Values represent the mean of three independent experiments (*n* = 3). CHL: chloramphenicol; RIF: rifampin; NDX: nalidixic acid; TET: tetracycline; COL: colistin. N.d.: Not determined.

Subsequently, log-phase *E. coli* ATCC 25922 cells (5 × 10^6^ CFU/mL) were incubated with each antimicrobial at its MIC value for 10 min. The amino acid analogue HPG was then added to the bacterial suspension and the incubation was prolonged for further 30 min. Subsequently bacteria were fixed, subjected to a click chemistry reaction, and analysed by flow cytometry to estimate the mean fluorescence intensity (MFI) values of the population (Figure 2, Supplementary Table S3).

We observed a significant fluorescence intensity decrease (75–90%) in bacterial samples treated with each of the three PrAMPs compared to the untreated bacteria (set as 100%). All three PrAMPs similarly inhibited HPG incorporation after 40 min of total incubation (Figure 2). As expected, chloramphenicol (CHL) caused an 80% reduction in fluorescence at its MIC. In contrast, rifampin (RIF) and nalidixic acid (NDX) did not affect fluorescence intensity, consistent with their distinct mechanisms of action targeting transcription and DNA replication, respectively (Figure 2).

The BONCAT protocol therefore effectively reported the inhibitory activity of PrAMPs on protein synthesis in living *E. coli* cells. We then verified whether this method could be applied also to other AMPs with unknown target with to define whether they are inhibitors of protein synthesis. Since most AMPs act on bacterial membranes, we investigated whether a combination of BONCAT and permeabilization assay was a valid approach to discriminate between genuine intracellular inhibitors and membrane-acting compounds.

**Figure 3:**
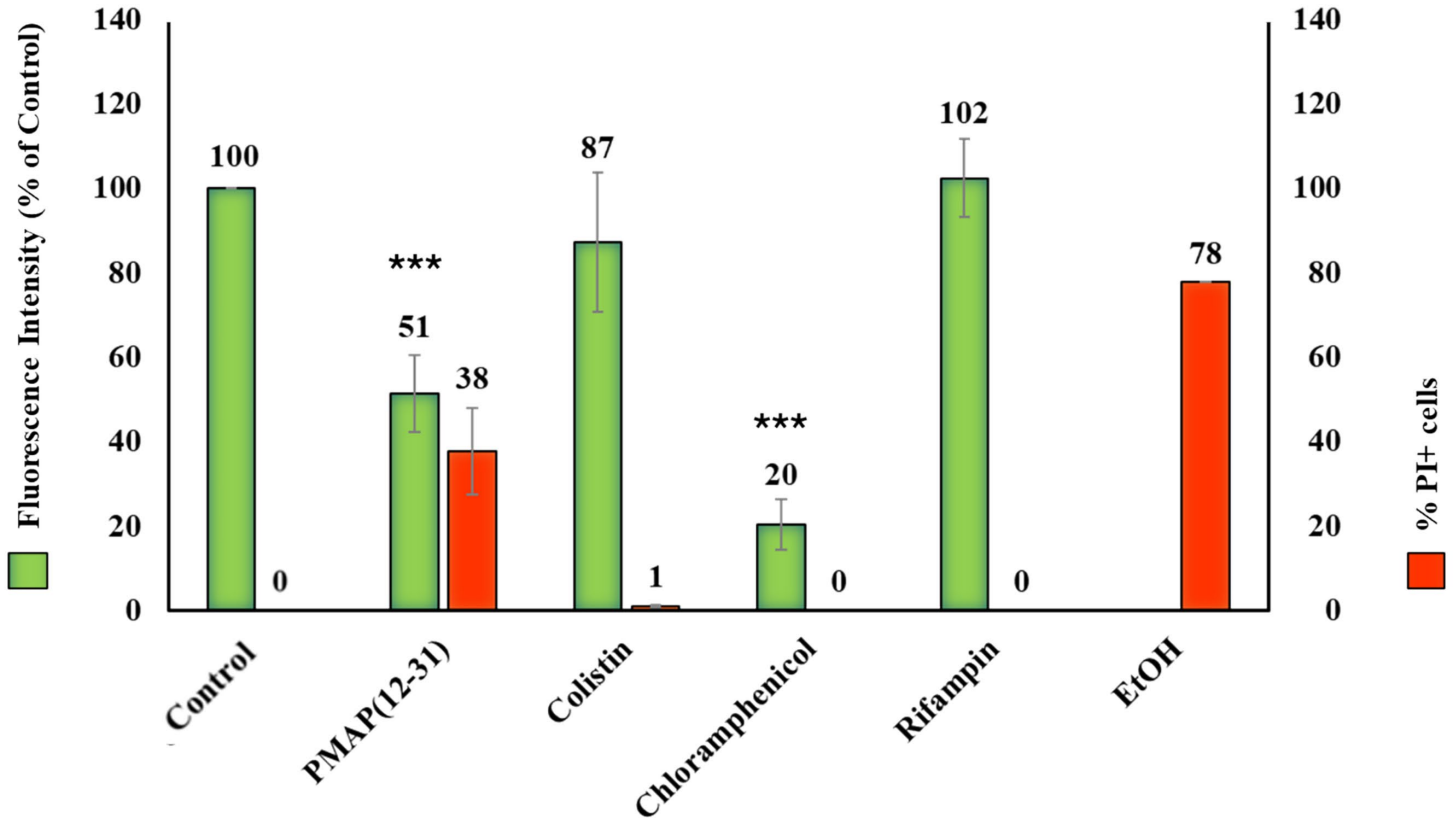
Flow cytometry analysis of *E. coli* cells treated with membranolytic peptides or antibiotics. Fluorescence (in green) is due to cells incubated 30 min with HPG, then fixed and subjected to click-chemistry with the fluorescent probe Alexa azide 488. Fluorescence percentages were recorded for cells treated with or without the indicated compounds for 10 min, followed by incubation with HPG for an additional 30 min. Each peptide or antibiotic was tested at its MIC concentration reported in Table 1. Cell permeabilization (in red) was assessed by incubating the bacteria with each compound at their MICs for 40 min. Ethanol was used as a positive control for membrane permeabilization. The results are expressed as the percentage of PI-positive cells compared to the total number of bacteria. Data represent the mean and standard deviation of at least three independent experiments (*n* ≥ 3). ***p < 0.001 compared with the untreated sample (one-way ANOVA test).

For this, we utilized two distinct membranolytic peptides: the alpha-helical PMAP (12–31), an active fragment derived from the known lytic AMP PMAP-36 (Biondi et al., 2023; Scheenstra et al., 2019) and the peptide antibiotic colistin, COL (Taglialegna, 2023). Analogous to the PrAMPs previously used, we determined MIC values and membrane permeabilization activity using the same medium and bacterial density described above (Table 1). Both colistin and PMAP(12–31) displayed a significant increase in MIC values (ranging from 8 to 32-fold) at higher bacterial concentrations under the conditions used (Supplementary Table S1). Quite surprisingly, colistin showed no membrane permeabilization at its MIC after 40 min of incubation. In contrast, PMAP(12–31), at its MIC, permeabilized 38% of the bacterial population. We then applied both colistin and PMAP(12–31) to the same BONCAT protocol used for PrAMPs, and for comparison, we used also chloramphenicol and rifampin as positive controls for direct protein synthesis and transcription inhibition, respectively. We observed that the AMP PMAP(12–31) determined a decrease in cell fluorescence (−49%) after 40 min of incubation concurrent to an increase of bacterial cell permeabilization (+38%) compared to the untreated control (Figure 3). In contrast, colistin, affected neither the integrity of bacterial membranes within the timeframe we applied nor decreased bacterial fluorescence, thus indicating even no indirect inhibition of protein synthesis (Figure 3). Both these cases suggest a positive correlation between the extent of cell permeabilization and a decrease in fluorescence due to indirect inhibition of protein synthesis. Therefore, a combined assay that includes both cell membrane permeabilization and BONCAT can distinguish a specific, primary inhibition of protein synthesis from the indirect effects of a generic metabolic failure, which occurs in fatally permeabilized cells.

**Figure 4:**
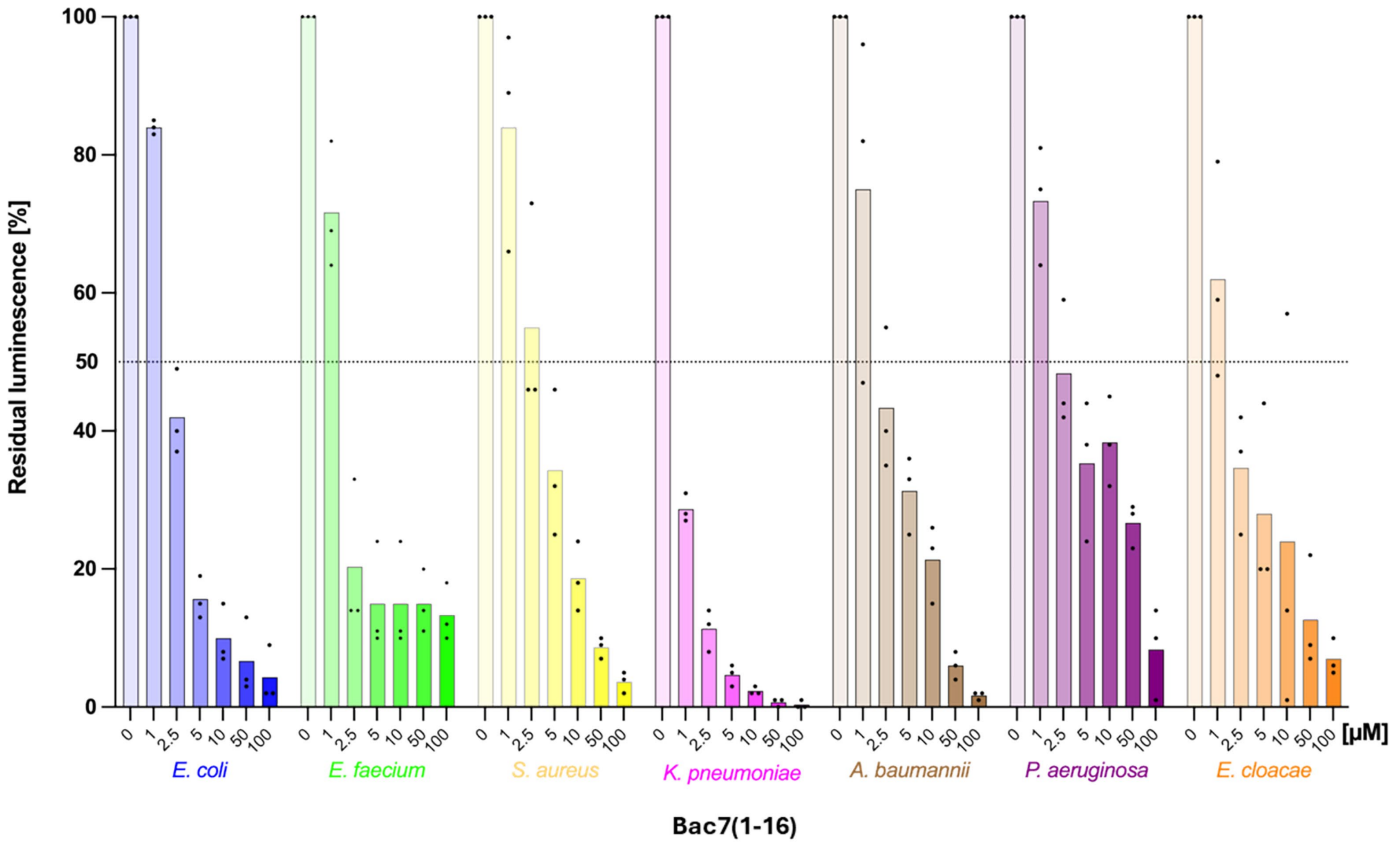
Inhibition of *in vitro* protein synthesis in different bacteria by Bac7(1–16). The assay was performed using the PURExpress system (NEB), supplemented with extracted and purified *E. coli* or distinct ESKAPE functional ribosomes. Samples treated with water (instead of the peptide) served as positive controls, with their efficacy set at 100%. Histograms represent the average of at least three independent experiments (*n* ≥ 3), with individual results indicated by black dots.

We then evaluated the applicability of this assay to bacterial species other than *E. coli*. To assess which of the ESKAPE pathogens could be most efficiently targeted by Bac7(1–16) in terms of their ability to block protein synthesis. We therefore performed an *in vitro* protein synthesis inhibition assay. Functional ribosomes extracted and purified from each of the ESKAPE pathogens were combined with the commercial purified *E. coli* translational machinery, which was deprived of its own ribosomes. This reconstituted a complete, functional translational machinery including the ribosomes derived from all the ESKAPE pathogens. *E. coli* ribosomes were also used for comparison. *In vitro* protein synthesis reactions were performed in the presence and in absence of Bac7(1–16), evaluating the productions (and activity) of firefly luciferase as reporter. The peptide impaired protein synthesis in all bacterial strains, albeit with different efficiency (Figure 4). We found that Bac7(1–16) at 5 μM inhibited 90% *K. pneumoniae* ribosomes, a value comparable to, of even exceeding, the inhibition observed for *E. coli* ribosomes in the same assay (Figure 4).

We repeated the BONCAT protocol with *K. pneumoniae* cells after confirming the strain’s ability to grow in supplemented M9 medium base devoid of amino acids (see Supplementary Figure S1). We also determined the MIC values for the same panel of PrAMPs against 5 × 10^6^ cells/mL *K. pneumoniae* ATCC 700603 cells in supplemented M9 medium. Overall, *K. pneumoniae* was susceptible to all PrAMPs, even though the MIC values of Bac7(1–16) and Bac7(1–35) were 4-fold higher than those for *E. coli,* while B7-005 displayed an overall comparable activity on both the bacterial species (Table 1). We also excluded any potential damaging effects on membranes at the MIC, to ensure any variation of the protein synthesis evaluated by BONCAT was a direct effect on the translation and not a downstream of membrane disruption (Table 1).

**Figure 5:**
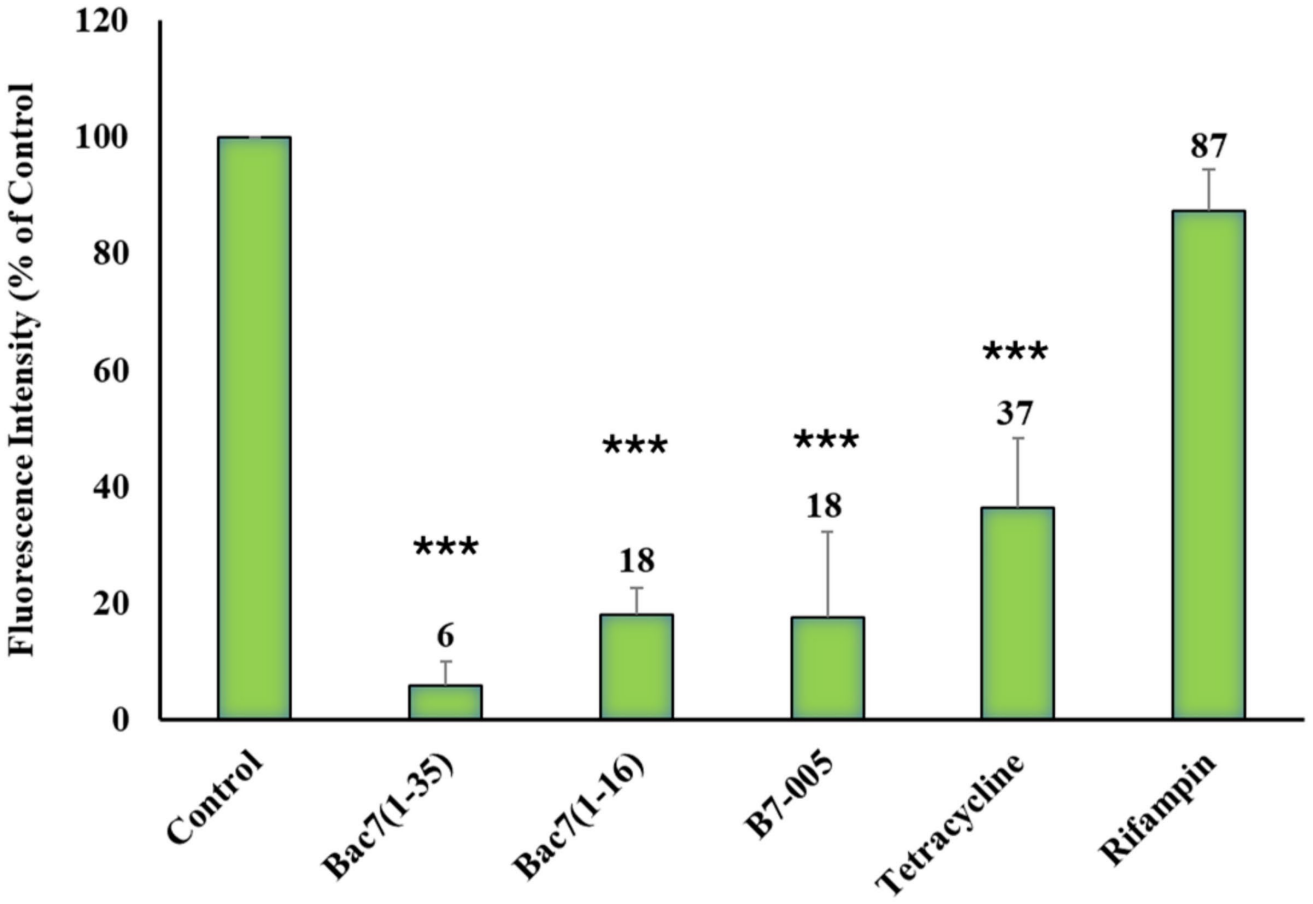
Flow cytometry analysis of HPG incorporation in *K. pneumoniae* cells treated with PrAMPs or antibiotics. Cells were pre-incubated for 10 min with the indicated compounds at their respective MICs (reported in the Table 1), followed by a 30-min incubation with the amino acid analogue HPG. After fixation, bacterial samples underwent a click chemistry reaction with the fluorescent probe Alexa Fluor azide 488 to label newly synthesized proteins. Fluorescence was measured and expressed as a percentage relative to untreated cells (Control) set as 100%. Data represent the mean and standard deviation of at least three independent experimental results (*n* ≥ 3). *** *p* < 0.001 compared with the untreated sample (one-way ANOVA test).

PrAMPs significantly inhibited incorporation in HPG in *K pneumoniae* (Figure 5), with inhibition percentages similar to those observed in *E. coli* (Figure 2). As expected, tetracycline, the antibiotic used as a control for protein synthesis inhibition, caused a relevant reduction (−65%) in fluorescence at its MIC. These results indicate that PrAMPs are at least as effective as tetracycline at inhibiting protein synthesis in *K. pneumoniae*. In contrast, rifampin did not affect fluorescence (Figure 5), consistent with its mechanism of action targeting RNA transcription while not also affecting downstream protein synthesis within a short timeframe. Therefore, we confirmed the effectiveness of BONCAT in verifying the impact of PrAMPs on protein synthesis in another clinically relevant pathogen besides the bacterial model *E. coli*. Furthermore, this study provides the first evidence that the tested mammalian PrAMPs inhibit protein synthesis in living *K. pneumoniae* cells.

Subsequently, we utilized the BONCAT protocol to further investigate the mechanism of protein synthesis inhibition by PrAMPs. Specifically, we examined the kinetics of translation inhibition and whether the inhibitory effects of the peptides persisted after the direct exposure of the bacterial culture to the drugs. To this end, the assay was limited to PrAMPs Bac7(1–16) and Bac7(1–35) only, as their mode of action relies purely on the inhibition of protein synthesis, unlikely B7-005 (Mardirossian et al., 2020). *E. coli* cells were incubated with Bac7(1–35) and Bac7(1–16) for 10 or 30 min, as described above, and antibiotics were used as a comparison. Following the incubation with the antimicrobials, bacteria were pelleted to remove the drugs from the medium and then re-incubated for 30-min with HPG.

**Figure 6:**
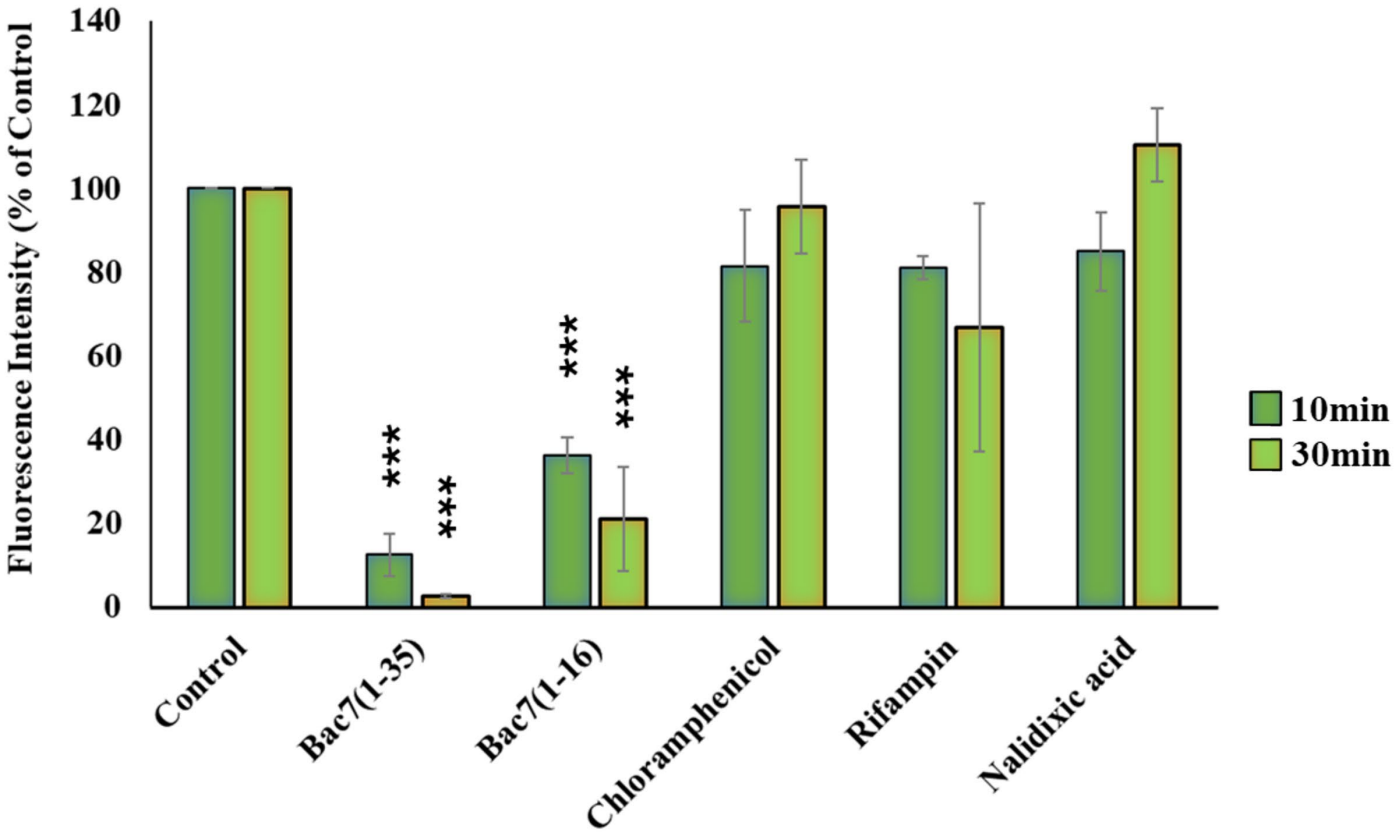
Flow cytometry analysis of *E. coli* protein synthesis inhibition after antimicrobial washout. The figure displays the fluorescence of newly synthesized proteins in *E. coli* after treatment with PrAMPs or control antibiotics. Cells were initially treated for 10 or 30 min at their minimum inhibitory concentration (MIC) before a complete washout of the compound. Protein synthesis was then assessed by incubating the cells with homopropargylglycine (HPG) for 30 min, followed by fixation and click chemistry with Alexa Fluor azide 488. Fluorescence was measured and expressed as a percentage relative to untreated cells (Control) set as 100%. Data, reflect the mean and standard deviation of fluorescence levels from a minimum of three independent experiments (*n* ≥ 3). ***p < 0.001 compared with the untreated sample (one-way ANOVA test).

The bacterial protein synthesis in cells treated with Bac7(1–35) and Bac7(1–16) remained significantly affected even after the washout (Figure 6). Under our experimental setting, Bac7(1–35) demonstrated very rapid activity, reducing almost completely protein synthesis, i.e.-88% after just 10 min and by −98% after 30 min of incubation. In contrast, chloramphenicol lost most of its inhibitory effects once removed from the medium (Figure 6), which implies a fundamental difference in its mechanism of action compared to the PrAMPs. An unexpected reduction in fluorescence of over 20% was however observed with rifampicin (Figure 6).

Unlike the antibiotic CHL, PrAMPs therefore maintained its inhibitory effect even after the peptide in the medium was washed out of the bacterial cell cultures. This led us to investigate whether this persistent inhibition by PrAMPs might also affects the bacterial growth rate.

To this end, *E. coli* cultures were incubated for 30 min with each of the PrAMPs as well as with control antibiotics at their respective MICs. The antimicrobial compounds were then washed out and bacterial growth was monitored by measuring optical density at 600 nm for 18 h.

**Figure 7:**
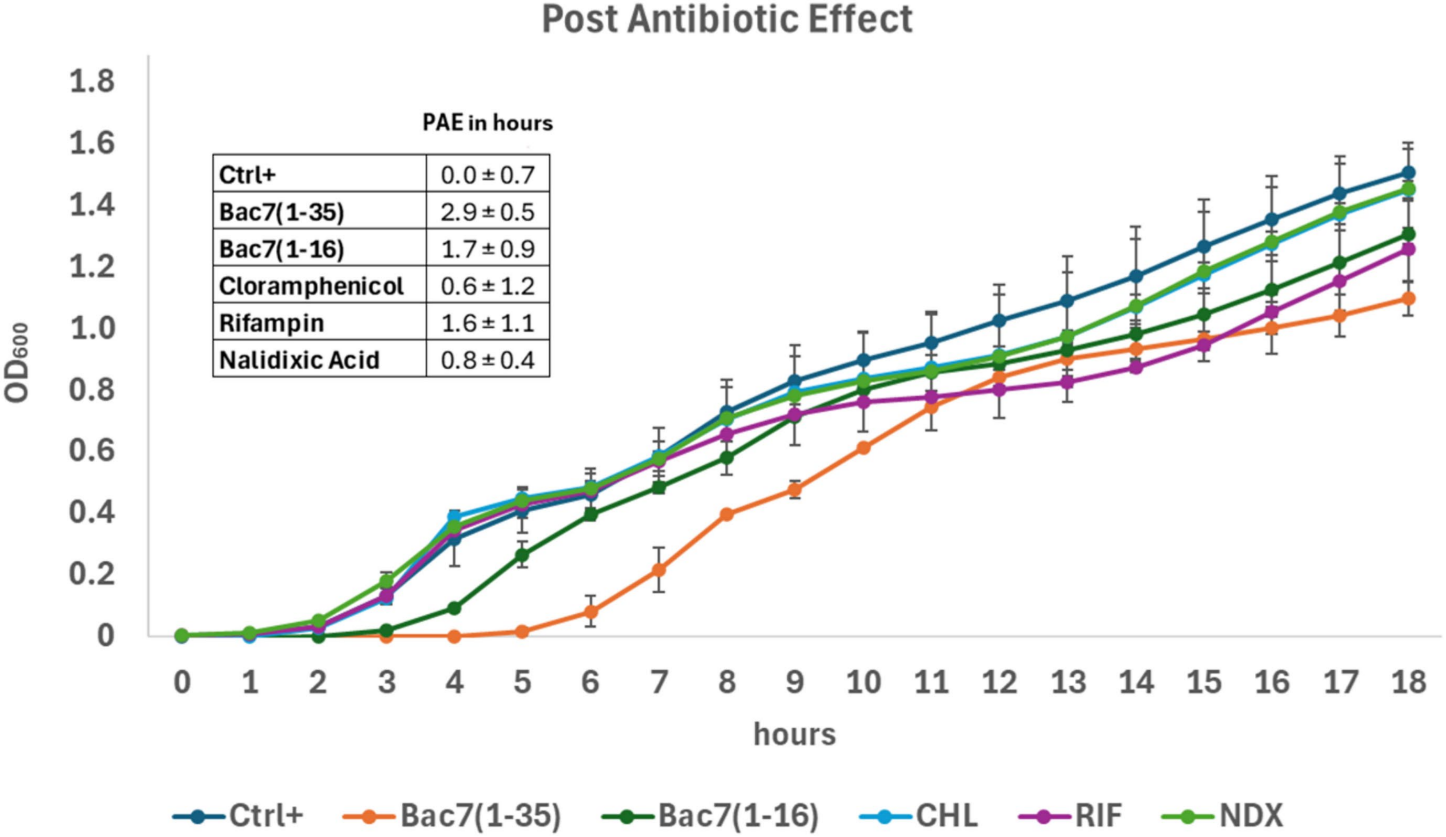
Bacterial cell growth kinetics assay to measure the post-antibiotic effect (PAE) of antimicrobial compounds. The PAE of different anti-microbial compounds on *E. coli* ATCC 25922, after a 30 min treatment followed by washout, was calculated by monitoring optical density at 600 nm every hour for 18 h and reported as Growth curves of treated and untreated cultures. The values (inbox) were calculated from the curves by interpolation (see Materials and Methods). PAE was calculated as the difference between the time required for a cell culture incubated with an antibacterial to reach the same OD600 of an untreated culture when it was at ½ of its logarithmic phase. Each peptide or antibiotic was tested at its MIC concentration. Data represent the mean and standard deviation of three independent experiments (*n* = 3).

The results indicate a temporary suppression of bacterial growth, manifesting as a delay in the growth of treated cells compared to untreated cells (Figure 7). This phenomenon is precisely defined as the Post-Antibiotic Effect (PAE) (Craig, 1993; Spangler et al., 1998), and PAE values calculated for different antimicrobial agents are shown in Figure 7. Specifically, Bac7(1–35) exhibited the longest-lasting PAE, preventing for 3 h the growth of treated bacteria compared to the untreated sample. Similarly, Bac7(1–16) showed a PAE, albeit shorter than Bac7(1–35) but comparable to other antibiotics such as rifampicin. In contrast, chloramphenicol demonstrated a PAE of about half an hour. This is in agreement with our protein synthesis inhibition assays, where the inhibitory effect of CHL was largely lost once removed from the medium. The PAE is also qualitatively reflected in the lag times observed for bacterial growth.

## Discussion

AMPs are a promising alternative to classical antibiotics. However, to consider them as therapeutic agents, it is crucial to understand their mechanisms of action (MoA). Over recent years, in fact, numerous methods have been developed, adapted, and refined to investigate the MoA of AMPs. Benfield and Henriques (2020), Schäfer and Wenzel (2020), Zhang et al. (2021). Nonetheless, it is important to acquire information on their MoA on living bacteria, which can be fundamentally different from their effect in simplified artificial models (Schäfer and Wenzel, 2020). Additionally, the MoA of an AMP can differ significantly among bacterial species (Di Stasi et al., 2025), underlining the need for specific characterization of each AMP/pathogen combination.

The primary objective of this work was to optimize and apply the BONCAT (Bioorthogonal Non-Canonical Amino Acid Tagging) method to investigate the *in vivo* intracellular mechanism of PrAMPs. This approach has been widely used in various fields, including eukaryotic cells (Dieterich et al., 2006) and bacterial studies to analyse mechanisms such as pathogenesis and virulence (Ignacio et al., 2021), quorum sensing (Bagert et al., 2016) and response to antibiotics (Babin et al., 2017). Our findings demonstrate that the BONCAT method is also an effective and reproducible tool for studying the intracellular mechanisms of AMPs that affect protein synthesis in living bacteria.

The protocol was initially validated using three well-characterized PrAMPs: Bac7(1–35), Bac7(1–16), and B7-005. These peptides have previously been shown to inhibit protein synthesis using complementary techniques, such as structural studies (Graf et al., 2017; Mardirossian et al., 2020; Seefeldt et al., 2016) or the incorporation of radioactive amino acids (Mardirossian et al., 2014). All the tested PrAMPs almost completely inhibited the synthesis of new proteins in living *E. coli*, which demonstrates that BONCAT is a suitable and specific tool for studying AMPs acting as protein synthesis inhibitors in bacteria.

We then demonstrated that this method can also be applied to bacterial species other than the model organism, *E. coli*. After testing the effect of PrAMPs on a cell-free ribosomal system, we selected *K. pneumoniae* due both to its clinical importance and because we established that its ribosomes are highly sensitive to PrAMP inhibition. The BONCAT results for *K. pneumoniae* were very similar to those observed in *E. coli*, suggesting that this method may be further extended to other susceptible bacteria able to grow in minimum media, thereby providing a versatile tool for MoA studies. Moreover, it represents the first observation of the MoA of PrAMPs on a living bacterial pathogen other than *E. coli*. Further investigation is warranted, especially in light of recent evidence indicating that molecular mechanism studies of antibacterial compounds on *E. coli* are not necessarily predictive of their action in other bacteria (Di Stasi et al., 2025).

A significant challenge in evaluating the mode of action of any antibacterial agent is distinguishing a primary, direct effect on a specific pathway or target, from the secondary consequences of cell death. For instance, an antibacterial agent acting primarily by compromising the cell membrane will eventually lead to a decline in protein synthesis as a result of ion influx, cellular content leakage, and cessation of energy production.

Our results with the lytic antimicrobial peptide PMAP(12–31) illustrate this point: it simultaneously decreased amino acid incorporation and caused membrane damage. In contrast, the normally membranolytic antibiotic colistin, which was unable to kill bacteria by lysis under the assay conditions we used, also did not affect protein synthesis. Crucially, the tested PrAMPs did not alter membrane permeability at protein synthesis-inhibitory concentrations. We showed this by combining the BONCAT protocol with a classic Propidium Iodide (PI) cytofluorimetric assay (Rosenberg et al., 2019), and this combination of techniques easily distinguishes a genuine ribosomal effect from an unspecific consequence of membrane disruption.

Overall, this combined method is well-suited for a variety of applications. It could be used to confirm the mode of action of a putative protein synthesis inhibitor or to investigate new antibacterial agents with unknown mechanisms. It therefore provides a robust platform to study the numerous AMPs for which an intracellular mode of action has been postulated but currently still lacks consolidated *in vivo* data (Le et al., 2017; Schäfer and Wenzel, 2020). Moreover, the BONCAT protocol could be applied for evaluating large numbers of potential antimicrobial agents and/or a broad range of target bacterial species, overcoming the complexity and limitations often associated with other techniques. A further optimization of BONCAT toward a high-throughput assay may foster the development of drugs targeting the bacterial protein synthesis.

Furthermore, BONCAT enabled us to precisely study the kinetics of PrAMPs inhibition of protein synthesis, an aspect not as thoroughly investigated using previous techniques. For instance, the killing kinetics of Bac7(1–35), Bac7(1–16) and B7-005 has previously been investigated using the viable colony count method. Results indicated that they determine a 10- to 100-fold reduction in viable *E. coli* cells within 1 h, at their minimum inhibitory concentrations (MICs), with Bac7(1–16) being significantly less rapid (Di Stasi et al., 2025; Podda et al., 2006), while our study reveals a more rapid action on their molecular target than on the whole microorganism. These peptides almost completely inhibited the incorporation of the amino acid analogue HPG into proteins after just 10 min of exposure. This demonstrates PrAMPs reach their molecular target on a timescale that is not very different from membranolytic peptides (Fantner et al., 2010) and comparable to, or shorter than that of traditional antibiotics (Harvey and Koch, 1980; Zhang et al., 2023). This finding shows that PrAMPs bind to ribosomes within minutes, a timeframe shorter than previously hypothesized for this class of molecules.

This work also highlighted a notable post-antibiotic effect (PAE), a phenomenon defined as the persistent suppression of bacterial growth after a brief exposure to an antimicrobial agent (Craig, 1993; Spangler et al., 1998). In the current study this effect was observed for the PrAMPs Bac7(1–35) and Bac7(1–16). The duration of the PAE *in vitro* is known to correlate with the antibiotic concentration, the duration of the exposure to the drug, and the bacterial species studied (Li et al., 1997). Our results showed a greater PAE for these PrAMPs compared to rifampicin, nalidixic acid and chloramphenicol, all used at their MICs. While rifampicin is known to induce a significant PAE (Chan et al., 2001) and nalidixic acid and chloramphenicol on the contrary generate only a moderate PAE (Athamna et al., 2004), our PrAMPs demonstrated a pronounced and sustained effect (see Figure 7). This finding is consistent with a previous study on ten different PrAMPs, including the full-length Bac7(1–60), used at their MIC for a one-hour exposure against *Escherichia coli*, *Klebsiella pneumoniae*, and *Pseudomonas aeruginosa* (Holfeld et al., 2018). In the current study, we also achieved a comparable growth arrest, lasting for several hours with 30 min exposure time. Our analysis revealed that after the removal of the peptides, protein synthesis remained inhibited for at least 30 min. It is plausible that a fraction of the peptides remains bound to their ribosomal targets even after the extracellular peptide concentration are reduced. A previous study demonstrated that Bac7(1–35) can accumulate at high concentrations within the *E. coli* cytoplasm (Mardirossian et al., 2014), making it highly likely that residual intracellular levels are sufficient to sustain inhibition of protein synthesis, thus prolonging the PAE. These observations have important pharmacodynamic implications, suggesting that PrAMPs can continue to inhibit bacterial protein synthesis even when their extracellular concentration falls below the effective threshold due to pharmacokinetic events (e.g., renal or hepatic clearance) upon a potential *in vivo* administration.

In addition, we observed that the efficacy of the PrAMP antibiotics used in this study, as measured by their MIC, did not decline when the bacterial density increased by 20-fold with respect to standard MIC procedures. In contrast, colistin and PMAP(12–31) exhibited an 8- to 64-fold increase in their MICs under the same conditions (see Supplementary Tables S1, S2). It is known that the efficacy of several antibacterial compounds decreases with increasing bacterial density (inoculum effect) (Loffredo et al., 2021), often due to reduced compound availability, due to effects such as sequestration by bacterial membranes. The increased total surface area of cell membranes, which are the principal targets of these compounds, results in more molecules binding, thereby requiring higher concentrations to achieve the threshold for the same antimicrobial effect (Loffredo et al., 2021). However, the PrAMPs tested in this study appear to bypass the limitations associated with the inoculum effect, confirming previous findings for Bac7(1–16) (Loffredo et al., 2021). The consistent MICs, regardless of the inoculum size, suggest that the mode of action of PrAMPs is not primarily influenced by membrane sequestration. It is plausible that at the MIC values, these PrAMPs are sufficient to saturate their intracellular binding sites, and that an increase in cell number does not significantly reduce the active peptide concentration required to inhibit protein synthesis, thus maintaining efficacy above the necessary threshold.

The combination of a long-lasting post-antibiotic effect (PAE) and the absence of an inoculum effect makes PrAMPs potentially advantageous in complex clinical contexts. For example, these characteristics could lead to reduced frequency of administration compared to traditional antibiotics and enable their effective use under conditions of high bacterial load.

In conclusion, this work contributes significantly to the functional characterization of PrAMPs. Moreover, our application, which combines permeabilization assays with BONCAT, establishes a suitable and powerful tool to study the mode of action of new antimicrobial compounds, including both AMPs and non-peptidic agents, even in the absence of preliminary information on their mechanism of action. The utility of this approach lies in its ability to foster the advancement of screening strategies for new antimicrobial agents, a necessity now more than ever in response to the global emergency of bacterial resistance.

## Data Availability

The original contributions presented in the study are included in the article/Supplementary material, further inquiries can be directed to the corresponding author.

## References

[ref1] Anderson R. C. Haverkamp R. G. Yu P.-L. (2004). Investigation of morphological changes to *Staphylococcus aureus* induced by ovine-derived antimicrobial peptides using TEM and AFM. FEMS Microbiol. Lett. 240, 105–110. doi: 10.1016/j.femsle.2004.09.027, 15500986

[ref2] Armas F. Di Stasi A. Mardirossian M. Romani A. A. Benincasa M. Scocchi M. (2021). Effects of Lipidation on a proline-rich antibacterial peptide. Int. J. Mol. Sci. 22:7959. doi: 10.3390/ijms22157959, 34360723 PMC8347091

[ref3] Athamna A. Athamna M. Medlej B. Bast D. J. Rubinstein E. (2004). In vitro post-antibiotic effect of fluoroquinolones, macrolides, β-lactams, tetracyclines, vancomycin, clindamycin, linezolid, chloramphenicol, quinupristin/dalfopristin and rifampicin on *Bacillus anthracis*. J. Antimicrob. Chemother. 53, 609–615. doi: 10.1093/jac/dkh130 14998982

[ref4] Babin B. M. Atangcho L. van Eldijk M. B. Sweredoski M. J. Moradian A. Hess S. . (2017). Selective proteomic analysis of antibiotic-tolerant cellular subpopulations in *Pseudomonas aeruginosa* biofilms. MBio 8:10.1128/mbio.01593-17. doi: 10.1128/mbio.01593-17 PMC5654934 29066549

[ref5] Bagert D. Kessel J. C. Sweredoski M. Feng L. Hess S. L. Bassler B. A. . (2016). Time-resolved proteomic analysis of quorum sensing in *Vibrio harveyi*. Chem. Sci. 7, 1797–1806. doi: 10.1039/C5SC03340C, 26925210 PMC4763989

[ref6] Benfield A. H. Henriques S. T. (2020). Mode-of-action of antimicrobial peptides: membrane disruption vs. intracellular mechanisms. Front. Med. Technol. 2:610997. doi: 10.3389/fmedt.2020.610997, 35047892 PMC8757789

[ref7] Benincasa M. Pelillo C. Zorzet S. Garrovo C. Biffi S. Gennaro R. . (2010). The proline-rich peptide Bac7(1-35) reduces mortality from *Salmonella typhimurium* in a mouse model of infection. BMC Microbiol. 10:178. doi: 10.1186/1471-2180-10-178, 20573188 PMC2896951

[ref8] Berthold N. Czihal P. Fritsche S. Sauer U. Schiffer G. Knappe D. . (2013). Novel Apidaecin 1b analogs with superior serum stabilities for treatment of infections by gram-negative pathogens. Antimicrob. Agents Chemother. 57, 402–409. doi: 10.1128/aac.01923-12, 23114765 PMC3535932

[ref9] Biondi B. de Pascale L. Mardirossian M. Di Stasi A. Favaro M. Scocchi M. . (2023). Structural and biological characterization of shortened derivatives of the cathelicidin PMAP-36. Sci. Rep. 13:15132. doi: 10.1038/s41598-023-41945-1, 37704689 PMC10499915

[ref10] Chan C.-Y. Au-Yeang C. Yew W.-W. Hui M. Cheng A. F. B. (2001). Postantibiotic effects of Antituberculosis agents alone and in combination. Antimicrob. Agents Chemother. 45, 3631–3634. doi: 10.1128/AAC.45.12.3631-3634.2001, 11709357 PMC90886

[ref11] Craig W. (1993). Pharmacodynamics of antimicrobial agents as a basis for determining dosage regimens. Eur. J. Clin. Microbiol. Infect. Dis. 12, S6–S8. doi: 10.1007/BF02389870 8477766

[ref12] Di Stasi A. Capolla S. Morici M. Bozzer S. Berger M. Pacor S. . (2025). Mechanistic divergence and differential antibacterial potency of the proline-rich antimicrobial peptide B7-005 across ESKAPE + E pathogens. Probiotics Antimicrob. Proteins. doi: 10.1007/s12602-025-10568-5 PMC12999614 40471535

[ref13] Dieterich D. C. Link A. J. Graumann J. Tirrell D. A. Schuman E. M. (2006). Selective identification of newly synthesized proteins in mammalian cells using bioorthogonal noncanonical amino acid tagging (BONCAT). Proc. Natl. Acad. Sci. 103, 9482–9487. doi: 10.1073/pnas.0601637103, 16769897 PMC1480433

[ref14] Fantner G. E. Barbero R. J. Gray D. S. Belcher A. M. (2010). Kinetics of antimicrobial peptide activity measured on individual bacterial cells using high speed AFM. Nat. Nanotechnol. 5, 280–285. doi: 10.1038/nnano.2010.29 20228787 PMC3905601

[ref15] Florin T. Maracci C. Graf M. Karki P. Klepacki D. Berninghausen O. . (2017). An antimicrobial peptide that inhibits translation by trapping release factors on the ribosome. Nat. Struct. Mol. Biol. 24, 752–757. doi: 10.1038/nsmb.3439, 28741611 PMC5589491

[ref16] Gagnon M. G. Roy R. N. Lomakin I. B. Florin T. Mankin A. S. Steitz T. A. (2016). Structures of proline-rich peptides bound to the ribosome reveal a common mechanism of protein synthesis inhibition. Nucleic Acids Res. 44, 2439–2450. doi: 10.1093/nar/gkw018, 26809677 PMC4797290

[ref17] Gera S. Kankuri E. Kogermann K. (2022). Antimicrobial peptides – unleashing their therapeutic potential using nanotechnology. Pharmacol. Ther. 232:107990. doi: 10.1016/j.pharmthera.2021.107990, 34592202

[ref18] Graf M. Mardirossian M. Nguyen F. Seefeldt A. C. Guichard G. Scocchi M. . (2017). Proline-rich antimicrobial peptides targeting protein synthesis. Nat. Prod. Rep. 34, 702–711. doi: 10.1039/c7np00020k, 28537612

[ref19] Graf M. Wilson D. N. (2019). “Intracellular antimicrobial peptides targeting the protein synthesis machinery” in Antimicrobial peptides: Basics for clinical application. ed. Matsuzaki K. (Singapore: Springer), 73–89. 10.1007/978-981-13-3588-4_6 30980354

[ref20] Hansen A. Schäfer I. Knappe D. Seibel P. Hoffmann R. (2012). Intracellular toxicity of proline-rich antimicrobial peptides shuttled into mammalian cells by the cell-penetrating peptide Penetratin. Antimicrob. Agents Chemother. 56, 5194–5201. doi: 10.1128/aac.00585-12, 22850523 PMC3457398

[ref21] Harvey R. J. Koch A. L. (1980). How partially inhibitory concentrations of chloramphenicol affect the growth of *Escherichia coli*. Antimicrob. Agents Chemother. 18, 323–337. doi: 10.1128/AAC.18.2.323, 6160809 PMC283991

[ref22] Hatzenpichler R. Scheller S. Tavormina P. L. Babin B. M. Tirrell D. A. Orphan V. J. (2014). Visualization of newly synthesized proteins in environmental microbes using amino acid tagging and click chemistry. Environ. Microbiol. 16, 2568–2590. doi: 10.1111/1462-2920.12436 24571640 PMC4122687

[ref23] Holfeld L. Knappe D. Hoffmann R. (2018). Proline-rich antimicrobial peptides show a long-lasting post-antibiotic effect on Enterobacteriaceae and *Pseudomonas aeruginosa*. J. Antimicrob. Chemother. 73, 933–941. doi: 10.1093/jac/dkx482 29309652

[ref24] Hsu C.-H. Chen C. Jou M.-L. Lee A. Y.-L. Lin Y.-C. Yu Y.-P. . (2005). Structural and DNA-binding studies on the bovine antimicrobial peptide, indolicidin: evidence for multiple conformations involved in binding to membranes and DNA. Nucleic Acids Res. 33, 4053–4064. doi: 10.1093/nar/gki725 16034027 PMC1179735

[ref25] Ignacio B. J. Bakkum T. Bonger K. M. Martin N. I. Kasteren S. I. (2021). Metabolic labeling probes for interrogation of the host–pathogen interaction. Org. Biomol. Chem. 19, 2856–2870. doi: 10.1039/D0OB02517H, 33725048

[ref26] Kiick K. L. Saxon E. Tirrell D. A. Bertozzi C. R. (2002). Incorporation of azides into recombinant proteins for chemoselective modification by the Staudinger ligation. Proc. Natl. Acad. Sci. USA 99, 19–24. doi: 10.1073/pnas.012583299, 11752401 PMC117506

[ref27] Krizsan A. Knappe D. Hoffmann R. (2015). Influence of the yjiL-mdtM gene cluster on the antibacterial activity of proline-rich antimicrobial peptides overcoming *Escherichia coli* resistance induced by the missing SbmA transporter system. Antimicrob. Agents Chemother. 59, 5992–5998. doi: 10.1128/aac.01307-15, 26169420 PMC4576061

[ref28] Krizsan A. Volke D. Weinert S. Sträter N. Knappe D. Hoffmann R. (2014). Insect-derived proline-rich antimicrobial peptides kill Bacteria by inhibiting bacterial protein translation at the 70 S ribosome. Angew. Chem. Int. Ed. 53, 12236–12239. doi: 10.1002/anie.201407145, 25220491

[ref29] Kuipers B. J. H. Gruppen H. (2007). Prediction of molar extinction coefficients of proteins and peptides using UV absorption of the constituent amino acids at 214 nm to enable quantitative reverse phase high-performance liquid chromatography−mass spectrometry analysis. J. Agric. Food Chem. 55, 5445–5451. doi: 10.1021/jf070337l, 17539659

[ref30] Lazzaro B. P. Zasloff M. Rolff J. (2020). Antimicrobial peptides: application informed by evolution. Science 368:eaau5480. doi: 10.1126/science.aau5480, 32355003 PMC8097767

[ref31] Le C.-F. Fang C.-M. Sekaran S. D. (2017). Intracellular targeting mechanisms by antimicrobial peptides. Antimicrob. Agents Chemother. 61:e02340-16. doi: 10.1128/AAC.02340-16, 28167546 PMC5365711

[ref32] Li R. C. Lee S. W. Kong C. H. (1997). Correlation between bactericidal activity and postantibiotic effect for five antibiotics with different mechanisms of action. J. Antimicrob. Chemother. 40, 39–45. doi: 10.1093/jac/40.1.39, 9249203

[ref33] Li W. Tailhades J. O’Brien-Simpson N. M. Separovic F. Otvos L. Hossain M. A. . (2014). Proline-rich antimicrobial peptides: potential therapeutics against antibiotic-resistant bacteria. Amino Acids 46, 2287–2294. doi: 10.1007/s00726-014-1820-1, 25141976

[ref34] Lindivat M. Larsen A. Hess-Erga O. K. Bratbak G. Hoell I. A. (2020). Bioorthogonal non-canonical amino acid tagging combined with flow cytometry for determination of activity in aquatic microorganisms. Front. Microbiol. 11:1929. doi: 10.3389/fmicb.2020.01929, 33013733 PMC7461810

[ref35] Loffredo M. R. Savini F. Bobone S. Casciaro B. Franzyk H. Mangoni M. L. . (2021). Inoculum effect of antimicrobial peptides. Proc. Natl. Acad. Sci. 118:e2014364118. doi: 10.1073/pnas.2014364118, 34021080 PMC8166072

[ref36] Magana M. Pushpanathan M. Santos A. L. Leanse L. Fernandez M. Ioannidis A. . (2020). The value of antimicrobial peptides in the age of resistance. Lancet Infect. Dis. 20, e216–e230. doi: 10.1016/S1473-3099(20)30327-3, 32653070

[ref37] Mahlapuu M. Björn C. Ekblom J. (2020). Antimicrobial peptides as therapeutic agents: opportunities and challenges. Crit. Rev. Biotechnol. 40, 978–992. doi: 10.1080/07388551.2020.1796576 32781848

[ref38] Mardirossian M. Barrière Q. Timchenko T. Müller C. Pacor S. Mergaert P. . (2018). Fragments of the nonlytic proline-rich antimicrobial peptide Bac5 kill *Escherichia coli* cells by inhibiting protein synthesis. Antimicrob. Agents Chemother. 62:18. doi: 10.1128/AAC.00534-18 PMC6105812 29844040

[ref39] Mardirossian M. Grzela R. Giglione C. Meinnel T. Gennaro R. Mergaert P. . (2014). The host antimicrobial peptide Bac71-35 binds to bacterial ribosomal proteins and inhibits protein synthesis. Chem. Biol. 21, 1639–1647. doi: 10.1016/j.chembiol.2014.10.009, 25455857

[ref40] Mardirossian M. Sola R. Beckert B. Valencic E. Collis D. W. P. Borišek J. . (2020). Peptide inhibitors of bacterial protein synthesis with broad Spectrum and SbmA-independent bactericidal activity against clinical pathogens. J. Med. Chem. 63, 9590–9602. doi: 10.1021/acs.jmedchem.0c00665, 32787108

[ref41] Mattiuzzo M. Bandiera A. Gennaro R. Benincasa M. Pacor S. Antcheva N. . (2007). Role of the *Escherichia coli* SbmA in the antimicrobial activity of proline-rich peptides. Mol. Microbiol. 66, 151–163. doi: 10.1111/j.1365-2958.2007.05903.x, 17725560

[ref42] Mukhopadhyay J. Sineva E. Knight J. Levy R. L. Ebright R. H. (2004). Antibacterial peptide microcin J25 (MCCJ25) inhibits transcription by binding within, and obstructing, the RNA polymerase secondary channel. Mol. Cell 14, 739–751. doi: 10.1016/j.molcel.2004.06.010 15200952 PMC2754415

[ref43] Oliveira Júnior N. G. Souza C. M. Buccini D. F. Cardoso M. H. Franco O. L. (2025). Antimicrobial peptides: structure, functions and translational applications. Nat. Rev. Microbiol. 23, 687–700. doi: 10.1038/s41579-025-01200-y, 40646173

[ref44] Podda E. Benincasa M. Pacor S. Micali F. Mattiuzzo M. Gennaro R. . (2006). Dual mode of action of Bac7, a proline-rich antibacterial peptide. Biochim. Biophys. Acta Gen. Subj. 1760, 1732–1740. doi: 10.1016/j.bbagen.2006.09.006, 17059867

[ref45] Raulf K. Koller T. O. Beckert B. Lepak A. Morici M. Mardirossian M. . (2025). The structure of the *Vibrio natriegens* 70S ribosome in complex with the proline-rich antimicrobial peptide Bac5(1–17). Nucleic Acids Res. 53:gkaf324. doi: 10.1093/nar/gkaf324, 40331629 PMC12056610

[ref46] Rosenberg M. Azevedo N. F. Ivask A. (2019). Propidium iodide staining underestimates viability of adherent bacterial cells. Sci. Rep. 9:6483. doi: 10.1038/s41598-019-42906-3, 31019274 PMC6482146

[ref47] Rostovtsev V. V. Green L. G. Fokin V. V. Sharpless K. B. (2002). A stepwise Huisgen cycloaddition process: copper(I)-catalyzed Regioselective “ligation” of Azides and terminal alkynes. Angew. Chem. Int. Ed. 41, 2596–2599. doi: 10.1002/1521-3773(20020715)41:14<2596::AID-ANIE2596>3.0.CO;2-4 12203546

[ref48] Roy R. N. Lomakin I. B. Gagnon M. G. Steitz T. A. (2015). The mechanism of inhibition of protein synthesis by the proline-rich peptide oncocin. Nat. Struct. Mol. Biol. 22, 466–469. doi: 10.1038/nsmb.3031, 25984972 PMC4456192

[ref49] Runti G. Benincasa M. Giuffrida G. Devescovi G. Venturi V. Gennaro R. . (2017). The mechanism of killing by the proline-rich peptide Bac7 (1–35) against clinical strains of *Pseudomonas aeruginosa* differs from that against other gram-negative bacteria. Antimicrob. Agents Chemother. 61:16. doi: 10.1128/aac.01660-16 PMC5365664 28137800

[ref50] Schäfer A.-B. Wenzel M. (2020). A how-to guide for mode of action analysis of antimicrobial peptides. Front. Cell. Infect. Microbiol. 10:540898. doi: 10.3389/fcimb.2020.540898, 33194788 PMC7604286

[ref51] Scheenstra M. R. van den Belt M. Tjeerdsma-van Bokhoven J. L. M. Schneider V. A. F. Ordonez S. R. van Dijk A. . (2019). Cathelicidins PMAP-36, LL-37 and CATH-2 are similar peptides with different modes of action. Sci. Rep. 9:4780. doi: 10.1038/s41598-019-41246-6, 30886247 PMC6423055

[ref52] Scocchi M. Mardirossian M. Runti G. Benincasa M. (2016). Non-membrane permeabilizing modes of action of antimicrobial peptides on bacteria. Curr. Top. Med. Chem. 16, 76–88. doi: 10.2174/1568026615666150703121009, 26139115

[ref53] Scocchi M. Tossi A. Gennaro R. (2011). Proline-rich antimicrobial peptides: converging to a non-lytic mechanism of action. Cell. Mol. Life Sci. 68, 2317–2330. doi: 10.1007/s00018-011-0721-7, 21594684 PMC11114787

[ref54] Seefeldt A. C. Graf M. Pérébaskine N. Nguyen F. Arenz S. Mardirossian M. . (2016). Structure of the mammalian antimicrobial peptide Bac7(1–16) bound within the exit tunnel of a bacterial ribosome. Nucleic Acids Res. 44, 2429–2438. doi: 10.1093/nar/gkv1545, 26792896 PMC4797285

[ref55] Seefeldt A. C. Nguyen F. Antunes S. Pérébaskine N. Graf M. Arenz S. . (2015). The proline-rich antimicrobial peptide Onc112 inhibits translation by blocking and destabilizing the initiation complex. Nat. Struct. Mol. Biol. 22, 470–475. doi: 10.1038/nsmb.3034, 25984971

[ref56] Shi J. Ross C. R. Chengappa M. M. Sylte M. J. McVey D. S. Blecha F. (1996). Antibacterial activity of a synthetic peptide (PR-26) derived from PR-39, a proline-arginine-rich neutrophil antimicrobial peptide. Antimicrob. Agents Chemother. 40, 115–121. doi: 10.1128/aac.40.1.115, 8787891 PMC163068

[ref57] Sneideris T. Erkamp N. A. Ausserwöger H. Saar K. L. Welsh T. J. Qian D. . (2023). Targeting nucleic acid phase transitions as a mechanism of action for antimicrobial peptides. Nat. Commun. 14:7170. doi: 10.1038/s41467-023-42374-4, 37935659 PMC10630377

[ref58] Sola R. Mardirossian M. Beckert B. De Luna L. S. Prickett D. Tossi A. . (2020). Characterization of cetacean proline-rich antimicrobial peptides displaying activity against ESKAPE pathogens. Int. J. Mol. Sci. 21, 1–17. doi: 10.3390/ijms21197367 PMC7582929 33036159

[ref59] Spangler S. K. Lin G. Jacobs M. R. Appelbaum P. C. (1998). Postantibiotic effect and Postantibiotic sub-MIC effect of levofloxacin compared to those of Ofloxacin, ciprofloxacin, erythromycin, azithromycin, and clarithromycin against 20 pneumococci. Antimicrob. Agents Chemother. 42, 1253–1255. doi: 10.1128/aac.42.5.1253, 9593160 PMC105793

[ref60] Subbalakshmi C. Sitaram N. (1998). Mechanism of antimicrobial action of indolicidin. FEMS Microbiol. Lett. 160, 91–96. doi: 10.1111/j.1574-6968.1998.tb12896.x, 9495018

[ref61] Taglialegna A. (2023). Reviving colistin. Nat. Rev. Microbiol. 21:411. doi: 10.1038/s41579-023-00907-0, 37173542

[ref62] Taniguchi M. Ochiai A. Kondo H. Fukuda S. Ishiyama Y. Saitoh E. . (2016). Pyrrhocoricin, a proline-rich antimicrobial peptide derived from insect, inhibits the translation process in the cell-free *Escherichia coli* protein synthesis system. J. Biosci. Bioeng. 121, 591–598. doi: 10.1016/j.jbiosc.2015.09.002, 26472128

[ref63] Tossi A. Gerdol M. Caporale A. Pacor S. Mardirossian M. Scocchi M. . (2024). Cathelicidins—a rich seam of antimicrobial peptides waiting for exploitation. Front. Drug Discov. 4:1458057. doi: 10.3389/fddsv.2024.1458057

[ref64] Travkova O. G. Moehwald H. Brezesinski G. (2017). The interaction of antimicrobial peptides with membranes. Adv. Colloid Interf. Sci. 247, 521–532. doi: 10.1016/j.cis.2017.06.001, 28606715

[ref65] Wang G. (2014). Human antimicrobial peptides and proteins. Pharmaceuticals 7, 545–594. doi: 10.3390/ph7050545, 24828484 PMC4035769

[ref66] Welch N. G. Li W. Hossain M. A. Separovic F. O’Brien-Simpson N. M. Wade J. D. (2020). (re)defining the proline-rich antimicrobial peptide family and the identification of putative new members. Front. Chem. 8:607769. doi: 10.3389/fchem.2020.607769 33335890 PMC7736402

[ref67] Yang B. Yang H. Liang J. Chen J. Wang C. Wang Y. . (2025). A review on the screening methods for the discovery of natural antimicrobial peptides. J. Pharmaceut. Anal. 15:101046. doi: 10.1016/j.jpha.2024.101046, 39885972 PMC11780100

[ref68] Zhang Y. Kepiro I. Ryadnov M. G. Pagliara S. (2023). Single cell killing kinetics differentiate phenotypic bacterial responses to different antibacterial classes. Microbiol. Spectr. 11:e0366722. doi: 10.1128/spectrum.03667-22, 36651776 PMC9927147

[ref69] Zhang Q.-Y. Yan Z.-B. Meng Y.-M. Hong X.-Y. Shao G. Ma J.-J. . (2021). Antimicrobial peptides: mechanism of action, activity and clinical potential. Military Med. Res. 8:48. doi: 10.1186/s40779-021-00343-2, 34496967 PMC8425997

