# Supplementary material for: Testing antimicrobial peptides inhibiting protein synthesis in living E. coli and K. pneumoniae using bio-orthogonal non-canonical amino-acid tagging
Source: Front Microbiol. 2025 Dec 16;16:1713216. doi: 10.3389/fmicb.2025.1713216 (PMC12748266; doi:10.3389/fmicb.2025.1713216)
Supplement: Supplementary file 1 [file Data_Sheet_1.docx]

**Supplementary materials:**

1. **Supplementary figure S1**

Growth curves of *E. coli* ATCC 25922 and *K. pneumoniae* ATCC 700603 in supplemented M9 medium

1. **Supplementary figure S2**

Representative flow cytometric image of fixed *E. coli* cells stained with propidium iodide (PI)

1. **Supplementary figure S3**

Colony growth assay in the presence of 16 µM HPG

1. **Supplementary table S1**

Comparison of MIC values for peptides and antibiotics against *E. coli* ATCC 25922 in different media at varying bacterial concentrations

1. **Supplementary table S2**

Comparison of MIC values for peptides and antibiotics against *K. pneumoniae* ATCC 700603 under different media and bacterial concentrations

1. **Supplementary table S3**

Raw data for mean fluorescence intensity (MFI) of HPG incorporation in E. coli cells treated with PrAMPs or antibiotics

1. **Supplementary figure S1**


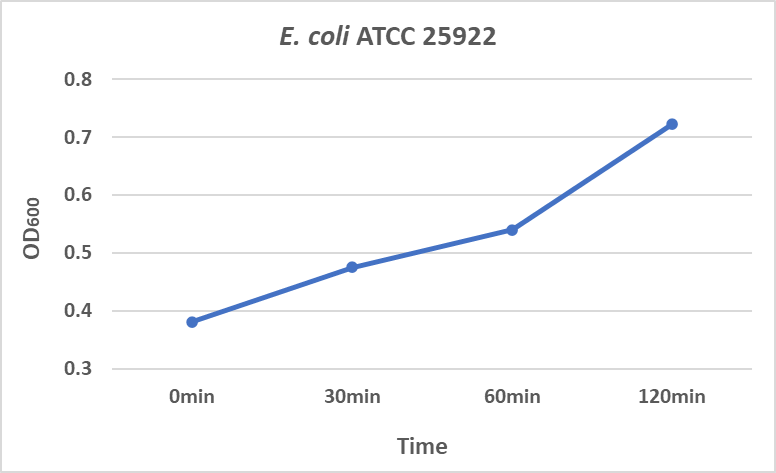

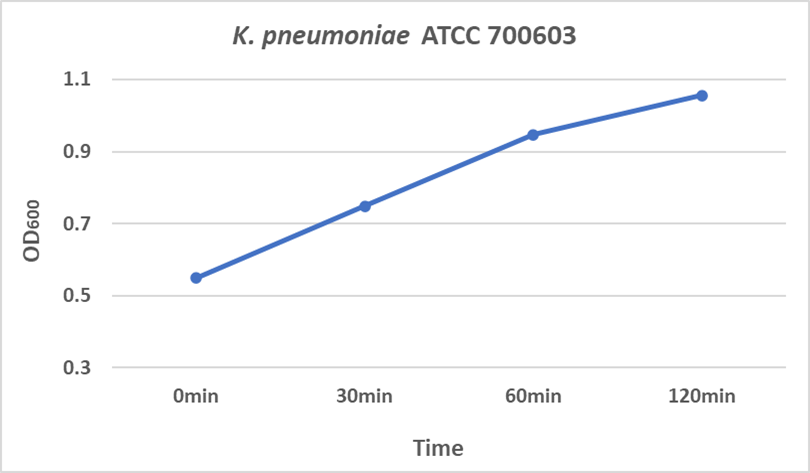


**Figure S1**: **growth curves of *E. coli* ATCC 25922 and *K. pneumoniae* ATCC 700603 in supplemented M9 medium**. Overnight cultures of *E. coli* ATCC 25922 (left panel) and K. pneumoniae (right panel), prepared as described in the Materials and Methods section, were resuspended in fresh supplemented M9 medium. Optical density at 600 nm (OD_600_) was measured at time zero and after 30 minutes, 1 hour, and 2 hours of incubation at 37 °C with shaking at 130 rpm. The supplemented M9 medium supported the growth of both bacterial strains, with OD₆₀₀ values approximately doubling within the first two hours, indicating active proliferation under the tested conditions.

1. **Supplementary figure S2**


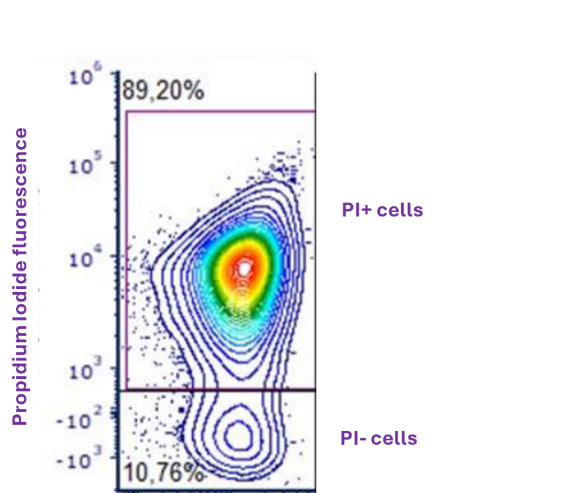


**Figure S2:** **Representative flow cytometric image of fixed *E. coli* cells stained with propidium iodide (PI)**. *E. coli* ATCC 25922 cells were permeabilized with EtOH, fixed with formaldehyde, and analysed by flow cytometry using PI (Rosemberg et al., 2019 as described in the Materials and Methods section. The gating strategy shown in the figure was established by analysing a control population of *E. coli* cells that were not subjected to fixation or permeabilization but incubated for 5 minutes with PI. In the sample shown, the gate for PI-positive (PI+) cells was set taking into account a sample without PI. Data acquisition was performed using Attune Flow Cytometer Software, and analysis was carried out using FCS Express Flow Cytometry software. Fixation and permeabilization resulted in approximately 90% of E. *coli* ATCC 25922 cells being PI+, indicating successful membrane permeabilization. Only PI+ cells were considered for the calculation of Alexa Fluor azide 488 MFI.

1. **Supplementary figure S3**


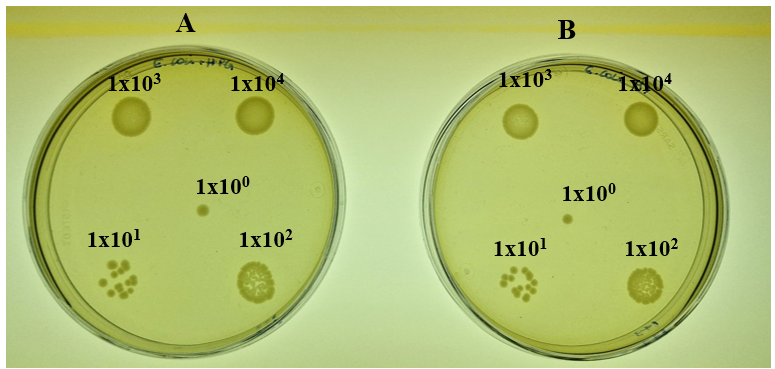


**Figure S3:** **Colony growth assay in the presence of 16 µM homopropargylglycine (HPG).** To assess potential toxicity to bacterial cells, *E. coli* ATCC 25922 (5 × 10⁶ CFU/mL), prepared as described in the Materials and Methods section, was incubated with 16 µM HPG (kiick et al., 2022) for 30 minutes in supplemented M9 medium, under conditions consistent with those used in BONCAT assays. Following incubation, serial 1:10 dilutions of the sample were performed in supplemented M9 medium, and each dilution was spotted onto MHB-agar plates. Plates were then incubated at 37 °C for 24 hours. Colony counts revealed no observable difference between the HPG-treated sample (panel A) and the untreated control (panel B), indicating that the methionine analogue HPG does not exert cytotoxic effects on *E. coli* under the tested conditions.

1. **Supplementary table S1**

**Table S1**: **Comparison of MIC values for peptides and antibiotics against *E. coli* ATCC 25922 in different media at varying bacterial concentrations**.

| **Compounds** | **MIC (µM)** | | |
| --- | --- | --- | --- |
|  | **Supplemented M9** | **Mueller Hinton** | |
|  | **5x10^6^CFU/mL** | **5x10^6^ CFU/mL** | **2,5x10^5^ CFU/mL** |
| Bac7(1-35) | 1 | 1-2 | 0,5 |
| Bac7(1-16) | 1 | 2 | 1 |
| B7-005 | 2-8 | 2-8 | 1 |
| COL | 16 | 4 | 0,5 * |
| PMAP(12-31) | 128 | 64 | 2 |
| CHL | 32 | 64 | 16 |

* Colistin (COL) at this bacteria concentration determined around 50% permeabilized cells (PI+) after 40 minutes of incubation

The MIC assays were performed in supplemented M9 medium or in MHB medium, as described in the Materials and Methods section, using final bacterial concentration of 2,5×10^5^ or 5×10^6^ CFU/well. Plates were incubated at 37 °C for 18 hours and sealed with parafilm to minimize evaporation. The values reported in the table represent the mode from at least three independent experiments. As expected (Loffredo et al., 2021), a 20-fold increase in cell density resulted in a dramatic rise in the MIC of PMAP(12-31) (Biondi et al., 2023) (32-fold) and a moderate increase in the MIC of colistin (COL) (Taglialegna et al., 2023) and chloramphenicol (CHL) (4-8-fold). In contrast minor changes (2-fold) were observed for PrAMPs. The medium used for MIC determination also influenced *E. coli* susceptibility, though to a lesser extent. Specifically, a 2-4-fold increase in MIC was observed for the lytic peptides colistin and PMAP(12-31) when tested in M9 medium compared to MHB.

1. **Supplementary table S2**

**Table S2**: **Comparison of MIC values for peptides and antibiotics against *K. pneumoniae* ATCC 700603 under different media and bacterial concentrations.**

| **Compounds** | **MIC (µM)** | |
| --- | --- | --- |
|  | **Supplemented M9** | **Mueller Hinton** |
|  | **5x10^6^CFU/mL** | **2,5x10^5^ CFU/mL** |
| Bac7(1-35) | 4 | 2 |
| Bac7(1-16) | 4 | 4 |
| B7-005 | 4 | 2 |
| COL | 16 | 1 |

The MIC assays were performed as described in the Materials and Methods section, using either the conditions set for the BONCAT assay (supplemented M9 medium and 5×10^6^ CFU/well) or standard conditions (MHB medium and 2,5×10^5^ CFU/well). Plates were incubated at 37 °C for 18 hours and sealed with parafilm to minimize evaporation. The values reported in the table represent the mode from at least three independent experiments. The 20-fold increase in cell density in the presence of supplemented M9 medium resulted in a 16-fold increase in the MIC of colistin, while only minor changes (1-2-fold) were observed for PrAMPs.

1. **Supplementary table S3**

**Table S3**: **Mean fluorescence intensity (MFI) of HPG incorporation in *E. coli* cells treated with PrAMPs or antibiotics**

| **Exp. n.** |  | **Control** | **Bac7(1-35)** | **Bac7(1-16)** | **B7-005** | **CHL** | **RIF** | **NDX** |
| --- | --- | --- | --- | --- | --- | --- | --- | --- |
| **1°** | Mean Fluorescence Intensity | **3654** |  | **580** |  | **953** |  | **3257** |
|  | % of control | 100 |  | 16 |  | 26 |  | 89 |
| **2°** | Mean Fluorescence Intensity | **1699** |  | **221** |  | **352** | **1559** | **1724** |
|  | % of control | 100 |  | 13 |  | 21 | 92 | 101 |
| **3°** | Mean Fluorescence Intensity | **2496** |  | **323** |  | **351** | **2598** | **2611** |
|  | % of control | 100 |  | 13 |  | 14 | 104 | 105 |
| **4°** | Mean Fluorescence Intensity | **2547** | **246** |  |  |  | **2547** |  |
|  | % of control | 100 | 10 |  |  |  | 100 |  |
| **5°** | Mean Fluorescence Intensity | **2861** | **263** | **468** |  |  |  | **3135** |
|  | % of control | 100 | 9 | 16 |  |  |  | 110 |
| **6°** | Mean Fluorescence Intensity | **2540** | **429** |  | **321** |  | **2890** |  |
|  | % of control | 100 | 17 |  | 13 |  | 114 |  |
| **7°** | Mean Fluorescence Intensity | **1957** | **368** | **352** |  |  |  | **2267** |
|  | % of control | 100 | 19 | 18 |  |  |  | 116 |
| **8°** | Mean Fluorescence Intensity | **1273** |  | **236** |  |  |  | **1402** |
|  | % of control | 100 |  | 19 |  |  |  | 110 |
| **9°** | Mean Fluorescence Intensity | **1493** |  | **350** |  |  |  | **1720** |
|  | % of control | 100 |  | 23 |  |  |  | 115 |
| **10°** | Mean Fluorescence Intensity | **1334** |  | **262** |  |  |  | **1424** |
|  | % of control | 100 |  | 20 |  |  |  | 107 |
| **11°** | Mean Fluorescence Intensity | **1661** |  | **417** |  |  |  |  |
|  | % of control | 100 |  | 25 |  |  |  |  |
| **12°** | Mean Fluorescence Intensity | **2446** |  | **488** | **695** |  |  |  |
|  | % of control | 100 |  | 20 | 28 |  |  |  |
| **13°** | Mean Fluorescence Intensity | **2423** |  |  | **657** |  |  |  |
|  | % of control | 100 |  |  | 27 |  |  |  |

We performed the BONCAT assay as described in the Materials and Methods section. The accompanying Table shows the raw mean fluorescence intensity (MFI) data obtained by flow cytometry across all experiments used to prepare the Figure 2. The MFI value of each sample (treated with antibiotics or PrAMPs or left untreated), was normalized and expressed as a percentage relative to the respective untreated control (% of control) within the same experimental run. The final data points presented graphically in the Figure 2 histogram of the main article represent the average of these normalized percentage values for each antimicrobial compound.
